# Genetic Variation in Toll-Like Receptor 5 and Colonization with Flagellated Bacterial Vaginosis-Associated Bacteria

**DOI:** 10.1128/IAI.00060-20

**Published:** 2021-02-16

**Authors:** Erin J. dela Cruz, Tina L. Fiedler, Congzhou Liu, Matthew M. Munch, Christina M. Kohler, Antoinette R. Oot, Jacqueline M. Wallis, Junhui Wang, Anna Frishman, Kristina Garcia, Andrew Wiser, Jennifer E. Balkus, Sujatha Srinivasan, Jonathan L. Golob, Laura K. Sycuro, Jeanne M. Marrazzo, Thomas R. Hawn, David N. Fredricks

**Affiliations:** a Medical Scientist Training Program, School of Medicine, University of Washington, Seattle, Washington, USA; b Molecular and Cellular Biology, School of Medicine, University of Washington, Seattle, Washington, USA; c Vaccine and Infectious Disease Division, Fred Hutchinson Cancer Research Center, Seattle, Washington, USA; d Department of Epidemiology, School of Public Health, University of Washington, Seattle, Washington, USA; e Division of Allergy and Infectious Diseases, Department of Medicine, University of Washington, Seattle, Washington, USA; f Department of Microbiology, Immunology & Infectious Diseases, University of Calgary, Calgary, Alberta, Canada; g Snyder Institute for Chronic Diseases, University of Calgary, Calgary, Alberta, Canada; h Division of Infectious Diseases, Department of Medicine, University of Alabama School of Medicine, Birmingham, Alabama, USA; University of California San Diego School of Medicine

**Keywords:** bacterial vaginosis, microbiome, Toll-like receptors, flagella

## Abstract

Bacterial vaginosis (BV) is a vaginal dysbiotic condition linked to negative gynecological and reproductive sequelae. Flagellated bacteria have been identified in women with BV, including *Mobiluncus* spp. and BV-associated bacterium-1 (BVAB1), an uncultivated, putatively flagellated species. The host response to flagellin mediated through Toll-like receptor 5 (TLR5) has not been explored in BV. Using independent discovery and validation cohorts, we examined the hypothesis that TLR5 deficiency—defined by a dominant negative stop codon polymorphism, rs5744168—is associated with an increased risk for BV and increased colonization with flagellated bacteria associated with BV (BVAB1, Mobiluncus curtisii, and Mobiluncus mulieris). TLR5 deficiency was not associated with BV status, and TLR5-deficient women had decreased colonization with BVAB1 in both cohorts. We stimulated HEK-hTLR5-overexpressing NF-κB reporter cells with whole, heat-killed *M. mulieris* or *M. curtisii* and with partially purified flagellin from these species; as BVAB1 is uncultivated, we used cervicovaginal lavage (CVL) fluid supernatant from women colonized with BVAB1 for stimulation. While heat-killed *M. mulieris* and CVL fluid from women colonized with BVAB1 stimulate a TLR5-mediated response, heat-killed *M. curtisii* did not. In contrast, partially purified flagellin from both *Mobiluncus* species stimulated a TLR5-mediated response *in vitro*. We observed no correlation between vaginal interleukin 8 (IL-8) and flagellated BVAB concentrations among TLR5-sufficient women. Interspecies variation in accessibility of flagellin recognition domains may be responsible for these observations, as reflected in the potentially novel flagellin products encoded by *Mobiluncus* species versus those encoded by BVAB1.

## Abstract (precis)

Bacterial vaginosis (BV) is a vaginal dysbiotic condition linked to negative gynecological and reproductive sequelae. Flagellated bacteria have been identified in women with BV, including *Mobiluncus* spp. and BV-associated bacterium-1 (BVAB1), an uncultivated, putatively flagellated species.

## INTRODUCTION

Bacterial vaginosis (BV) is a common vaginal dysbiosis, involving a bacterial community shift from one composed of a few species of lactobacilli to a diverse community of anaerobic and facultative bacteria with increased species richness and evenness (1). Nugent score is the gold standard for diagnosing BV, involving examination of Gram-stained vaginal fluid by a trained microscopist who identifies bacterial morphotypes associated with BV or with its absence (2). While Nugent scores of 7 to 10 are defined as bacterial vaginosis, Nugent scores of 9 or 10 are defined by the presence of *Mobiluncus* morphotypes, i.e., curved, Gram-negative rods. Vaginal *Mobiluncus* spp. have been associated with host epithelial cells, flagellar structures observed using electron microscopy (3), and motility observed on wet mounts (4, 5). Using molecular methods, our group found that many of the bacterial cells classified as *Mobiluncus* morphotypes on Gram stains were BV-associated bacterium 1 (BVAB1) (6), an uncultivated member of the order *Clostridiales* (7) with high specificity for BV (8). Recently, a BVAB1 metagenome-assembled genome (MAG) was published (9), which demonstrated the presence of flagellar genes. Altogether, these studies raise the possibility that these flagellated species, which have been correlated with spontaneous preterm birth (9, 10), are able to use directed motility to ascend into the upper genital tract. Few other vaginal bacteria are known to produce flagella.

Although the involvement of flagellated organisms in BV has been established for over 30 years (4, 5, 11), vaginal innate immune responses to flagellin have not been evaluated. Toll-like receptor 5 (TLR5) responds to conserved regions located in the N and C termini of the flagellin monomer (12–15). TLR5 is strongly expressed by vaginal, ectocervical, and endocervical epithelium (16, 17). The TLR5 single nucleotide polymorphism (SNP) rs5744168 encodes a C-to-T transition at base pair 1174, which changes an arginine to a stop codon at amino acid 392 (TLR5-392^STOP^). CT heterozygotes, present in about 5 to 10% of the population, have deficient signaling in response to extracellular flagellin stimulation. The TT genotype is uncommon, and rigorous comparison of signaling levels between CT and TT individuals has not been performed. However, the blunted signaling of CT heterozygotes is consistent with a dominant negative genetic model wherein a stop codon mutation in TLR5 (referred to as TLR5 deficiency) abolishes TLR5 signaling (18) and is associated with increased susceptibility to infection with some flagellated organisms (18–20). TLR5 deficiency was associated with increased risk of ulcerative colitis in an Indian cohort (21) but also with decreased prevalence of Crohn’s disease in an Ashkenazi Jewish subpopulation in a U.S.-based study (22), suggesting a complex relationship between TLR5-mediated immune responses and clinical outcomes involving the microbiota.

We hypothesized that TLR5 deficiency would be associated with increased risk of clinically and microbiologically defined BV, as well as increased colonization with the flagellated BV-associated bacteria (BVAB) Mobiluncus curtisii, Mobiluncus mulieris, and BVAB1. Contrary to our hypothesis, we found no relationship between TLR5 deficiency and BV, and we also found that TLR5 deficiency is associated with decreased colonization with *M. mulieris* and BVAB1 but not with differences in *M. curtisii* colonization. We probed this relationship further, finding that cellular products of BVAB1 and flagellin from *M. mulieris* and *M. curtisii* stimulate a TLR5-specific innate immune response. We predicted this would lead women with TLR5 sufficiency to have higher concentrations of the chemokine interleukin 8 (IL-8) with increasing concentrations of these flagellated BVAB. IL-8 was selected because it is induced by flagellin, regulated by TLR5, secreted by HEK293 cells, and present in vaginal fluid from women with BV. Contrary to expectations, IL-8 was not correlated with absolute abundance of BVAB1, *M. mulieris*, or *M. curtisii*. Differences in flagellin composition among bacteria may explain differences in TLR5 responses noted here.

## RESULTS

### Cohort characteristics.

A longitudinal discovery cohort was composed of 213 women enrolled from the community in Seattle, WA, of whom 124 were BV negative by Amsel’s clinical criteria (123 were BV negative by Nugent score) at enrollment. As our subsequent analyses were based on a subset of 189 with resolved TLR5 rs5744168 genotypes, we present the demographics of this subset here (Table 1). Cumulative incidence of BV among the BV-negative women in the discovery cohort was 36/113 (32%) by Amsel’s criteria and 35/111 (32%) by Nugent score. Concordant with previous studies (23), a history of BV was more common among participants with BV at enrollment.

**TABLE 1:** Discovery cohort demographics

Characteristic	BV^−^ (*n* = 113)^*a*^		BV^+^ (*n* = 76)^*a*^		Total (*n* = 189)		*P*_χ2_^*b*^
	No.	%	No.	%	No.	%	
Race							
AIAN/NHPI^*c*^	6	5	1	1	7	4	0.13
Asian	2	2	1	1	3	2	
African-American	28	25	32	42	60	32	
Caucasian	65	58	38	50	103	54	
Other	11	10	4	5	15	8	
Two or more races	1	1	0	0	1	1	
Ethnicity							
Hispanic	8	7	5	7	13	7	0.91
Non-Hispanic	91	81	61	80	152	80	
Refused to report	14	12	10	13	24	13	
Age (yr)							
18–30	52	46	38	50	90	48	0.15
31–40	29	26	25	33	54	29	
41–50	31	27	11	14	42	22	
Refused to report	1	1	2	3	3	2	
Hormonal contraception							
Yes	37	33	26	34	63	33	0.96
No	76	67	50	66	126	67	
Refused to report	0	0	0	0	0	0	
History of BV							
Yes	69	61	64	84	133	70	<0.01
No	41	36	10	13	51	27	
Refused to report	3	3	2	3	5	3	

a Bacterial vaginosis diagnosed by Amsel's criteria.

b *P*_χ2_, *P* value by Chi-square test.

c AIAN/NHPI, American Indian, Alaska Native, Native Hawaiian, Pacific Islander.

We compared the demographic and baseline clinical characteristics of the women whose genotypes were resolved compared to those whose genotypes we were unable to resolve (Table S1). In general, the two groups had similar composition by Nugent score, ethnicity, age, use of hormonal contraception, and history of BV. However, racial composition differed between the two groups (*P* = 0.03), with more African-American and fewer Caucasian women having unresolved genotypes.

The validation cohort was composed of 45 BV-negative and 66 BV-positive women (59% BV positive by Amsel’s criteria) (Table 2). Similar patterns were seen in this cohort with regard to race, ethnicity, and history of BV, with a wider age range of premenopausal women (ages 18 to 59), and fewer women reporting use of hormonal contraceptives (21%).

**TABLE 2:** Validation cohort enrollment demographics

Characteristic	BV^−^ (*n* = 45)^*a*^		BV^+^ (*n* = 66)^*a*^		Total (*n* = 111)		*p*_χ2_^*b*^
	No.	%	No.	%	No.	%	
Race							
AIAN/NHPI^*c*^	0	0	2	3	2	2	0.01
Asian	1	2	5	8	6	5	
African-American	9	20	27	41	36	32	
Caucasian	29	64	21	32	50	45	
Other	6	13	8	12	14	13	
Two or more races	0	0	3	5	3	3	
Ethnicity							
Hispanic	5	11	5	8	10	9	0.75
Non-Hispanic	36	80	45	68	81	73	
Refused to report	4	9	16	24	20	18	
Age (yr)							
18–30	24	53	40	61	64	58	0.25
31–40	9	20	12	18	21	19	
41–50	7	16	9	14	16	14	
51–60	5	11	2	3	7	6	
Refused to report	0	0	3	5	3	3	
Hormonal contraception							
Yes	12	27	11	17	23	21	0.35
No	32	71	51	77	83	75	
Refused to report	1	2	4	6	5	5	
History of BV							
Yes	24	53	50	76	74	67	0.01
No	21	47	14	21	35	32	
Refused to report	0	0	2	3	2	2	

a Bacterial vaginosis diagnosed by Amsel's criteria.

b *P*_χ2_, *P* value by Chi-square test.

c AIAN/NHPI, American Indian, Alaska Native, Native Hawaiian, Pacific Islander.

### TLR5 deficiency is not associated with clinically or microbiologically defined BV status.

**FIG 1:**
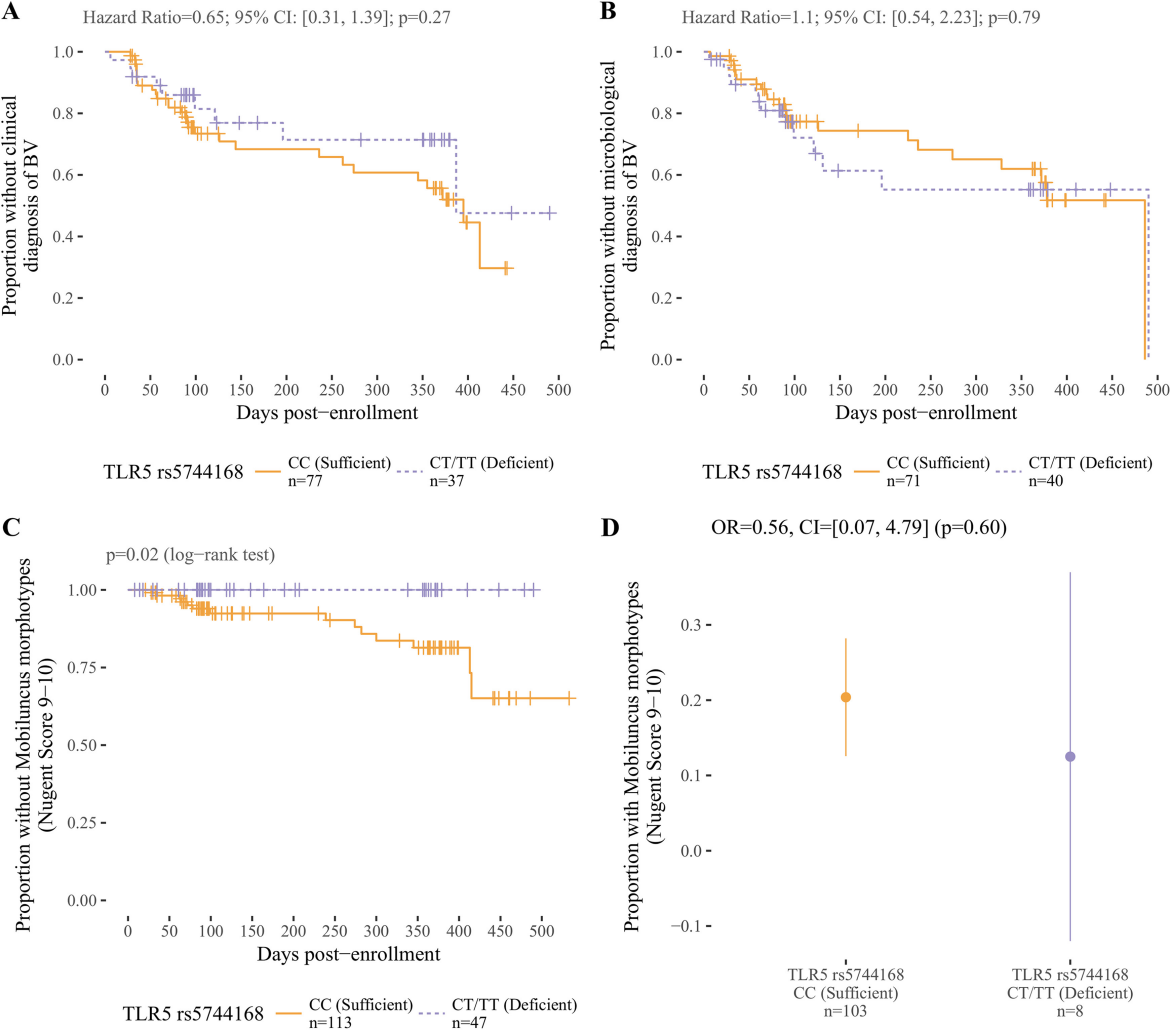
TLR5 deficiency is associated with decreased risk of having BV with the presence of *Mobiluncus* morphotypes. (A to C) Survival curves and results of Cox proportional hazards regression comparing time to first episode of BV associated with a clinic visit among TLR5-deficient (purple dashed line) versus TLR5-sufficient (orange solid line) women in the longitudinal discovery cohort. (A) TLR5-deficient and -sufficient women are at similar risk of clinically defined BV (Amsel’s criteria). (B) TLR5-deficient and -sufficient women are at similar risk of microbiologically defined BV (Nugent criteria). (C) Survival curve depicting the proportion without clinic-associated BV associated with the presence of *Mobiluncus* morphotypes. Of 47 TLR5-deficient women, none were observed to have *Mobiluncus* morphotypes at a follow-up clinic visit, compared to 14 of 113 TLR5-sufficient women (*P* = 0.02; log-rank test). (D) TLR5-deficient women in the validation cohort showed no significant difference in prevalence of *Mobiluncus* morphotypes. Dots and lines represent proportions of participants diagnosed with BV and 95% confidence intervals (CI), respectively. OR, odds ratio.

We hypothesized that TLR5 deficiency is associated with increased risk of both clinically and microbiologically defined BV. Contrary to our hypothesis, TLR5 deficiency was not associated with risk of incident BV in the discovery cohort or odds of BV in the validation cohort (Fig. 1A and B). Furthermore, TLR5 deficiency did not correlate with the following characteristics associated with BV: race, age, hormonal contraception, and self-reported history of BV (Table 3 [discovery cohort] and Table 4 [validation cohort]).

**TABLE 3:** Discovery cohort allele frequency

Characteristic	No. with TLR5 rs5744168 allele			Total no.	*P*_χ2_^*a*^
	CC^*b*^	CT^*c*^	TT^*c*^		
Total	138	47	4	189	
Race					
AIAN/NHPI^*d*^	6	1	0	7	0.45
Asian	2	1	0	3	
African-American	47	12	1	60	
Caucasian	72	28	3	103	
Other	11	4	0	15	
Two or more races	0	1	0	1	
Age (yr)					
18–30	66	22	2	90	0.76
31–40	39	15	0	54	
41–50	30	10	2	42	
Refused to report	3	0	0	3	
Hormonal contraception					
Yes	45	16	2	63	0.86
No	93	31	2	126	
Refused to report	0	0	0	0	
History of BV					
Yes	98	31	4	133	0.30
No	35	16	0	51	
Refused to report	5	0	0	5	

a *P* value comparing demographics in TLR5-sufficient versus TLR5-deficient women.

b TLR5 sufficient.

c TLR5 deficient.

d AIAN/NHPI, American Indian, Alaska Native, Native Hawaiian, Pacific Islander.

**TABLE 4:** Validation cohort allele frequency

Characteristic	No. with TLR5 rs5744168 allele				Total no.	*P*_χ2_^*a*^
	CC^*b*^	CT^*c*^	TT^*c*^	NR^*d*^		
Total	103	8	0	0	111	
Race						
AIAN/NHPI^*e*^	2	0	0	0	2	0.63
Asian	6	0	0	0	6	
African-American	35	1	0	0	36	
Caucasian	44	6	0	0	50	
Other	10	4	0	0	14	
Two or more races	3	0	0	0	3	
Age (yr)						
18–30	61	3	0	0	64	0.23
31–40	17	4	0	0	21	
41–50	15	1	0	0	16	
51–60	7	0	0	0	7	
Refused to report	3	0	0	0	3	
Hormonal contraception						
Yes	23	0	0	0	23	0.27
No	75	8	0	0	83	
Refused to report	5	0	0	0	5	
History of BV						
Yes	67	7	0	0	74	0.40
No	34	1	0	0	35	
Refused to report	2	0	0	0	2	

a *P* value comparing demographics in TLR5-sufficient versus TLR5-deficient women.

b TLR5 sufficient.

c TLR5 deficient.

d NR, genotype was unable to be resolved or was not obtained.

e AIAN/NHPI, American Indian, Alaska Native, Native Hawaiian, Pacific Islander.

To explore whether TLR5 deficiency could specifically impact colonization with *Mobiluncus* morphotypes identified in Nugent scoring of Gram stains, we performed an exploratory analysis examining whether TLR5 deficiency was associated with vaginal fluid Nugent scores of 9 or 10 (which are associated with *Mobiluncus* morphotypes). Strikingly, in the discovery cohort, women with TLR5 deficiency were less likely to have *Mobiluncus* morphotypes on vaginal fluid Gram stains (Fig. 1C). In a survival analysis examining time to first Nugent score of 9 or 10, no TLR5-deficient women were observed at a clinic visit to have a Nugent score of 9 or 10 in 24.8 person-years of follow-up time, compared with 14 Nugent scores of 9 or 10 observed in 61.0 person-years of follow-up in TLR5-sufficient women (*P* = 0.02, log-rank test). In the validation cohort, 1 of 8 (13%) TLR5-deficient versus 21 of 103 (20%) TLR5-sufficient women had Nugent scores of 9 or 10 (Fig. 1D). However, our logistic regression model identified no association between TLR5 genotype and BV diagnosis (*P* = 0.60).

### TLR5 deficiency is associated with decreased colonization with BVAB1 and *M. mulieris*.

**FIG 2:**
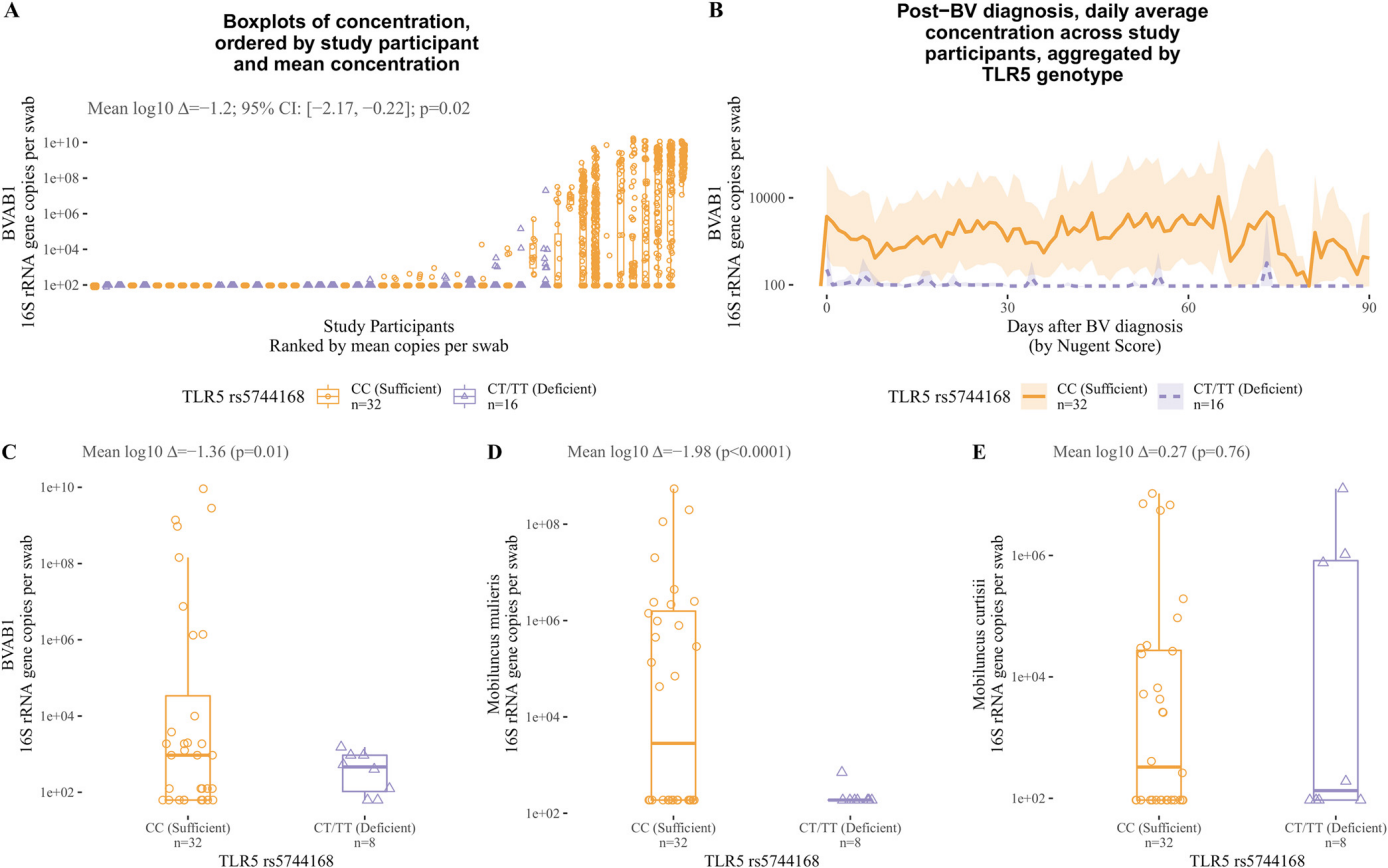
TLR5 deficiency is associated with decreased colonization with BVAB1 and *M. mulieris*. (A) Discovery cohort colonization with BVAB1 in TLR5-deficient cases and matched TLR5-sufficient controls, divided into separate box plots with overlaid scatterplots for each study participant to show within-subject variability. Women with TLR5 deficiency had an average of 1.2-log_10_ fewer BVAB1 16S rRNA gene copies per swab than matched controls (*P* = 0.02; analysis of variance [ANOVA]). (B) Discovery cohort colonization with BVAB1 in TLR5-deficient cases and matched TLR5-sufficient controls, depicted as a daily, moving average aggregated by TLR5 deficiency versus sufficiency (shading depicts 95% bootstrapped confidence interval). At the vast majority of time points after BV diagnosis, the confidence intervals of mean colonization with BVAB1 in TLR5-deficient and sufficient participants do not overlap, providing evidence that TLR5-deficient women have lower concentrations of BVAB1. (C and D) In the validation cohort, TLR5-deficient women had an average of 1.36 log_10_ fewer BVAB1 16S rRNA gene copies per swab than TLR5-sufficient women (C) and 1.98 log_10_ fewer *M. mulieris* 16S rRNA gene copies per swab (D). (E) In the validation cohort, TLR5-sufficient and -deficient women had similar colonization with *M. curtisii*.

While our original hypothesis posited that concentrations of BVAB1 and *Mobiluncus* spp. are increased in TLR5-deficient women, the decreased incidence and prevalence of *Mobiluncus* morphotypes by Gram stain suggested the opposite. To probe this further, BVAB1 colonization was measured using quantitative PCR (qPCR) in matched TLR5-deficient and -sufficient individuals in the discovery cohort (Fig. S1). We found that BVAB1 concentrations were significantly decreased in women with TLR5 deficiency (Fig. 2A and B). As expected, nonflagellated vaginal bacteria were not significantly different between TLR5-sufficient and -deficient discovery cohort participants (Fig. S2). Specifically, we examined *Gardnerella* spp. and Lactobacillus crispatus as representative, nonflagellated vaginal bacteria associated with BV and its absence, respectively. The correlation between TLR5 deficiency and decreased BVAB1 colonization was replicated in the validation cohort (Fig. 2C).

In matched TLR5-deficient and -sufficient participants in the validation cohort (Table S2), TLR5-deficient women had significantly decreased concentrations of *M. mulieris* (Fig. 2D) and no significant differences in *M. curtisii* (Fig. 2E). Concentrations of *Mobiluncus* spp. were not assessed in the discovery cohort. As in the discovery cohort, *Gardnerella* spp. and L. crispatus were not significantly different between TLR5-deficient and -sufficient women in the validation cohort (Fig. S3).

### Predicted TLR5 agonism based on flagellin amino acid sequences.

**FIG 3:**
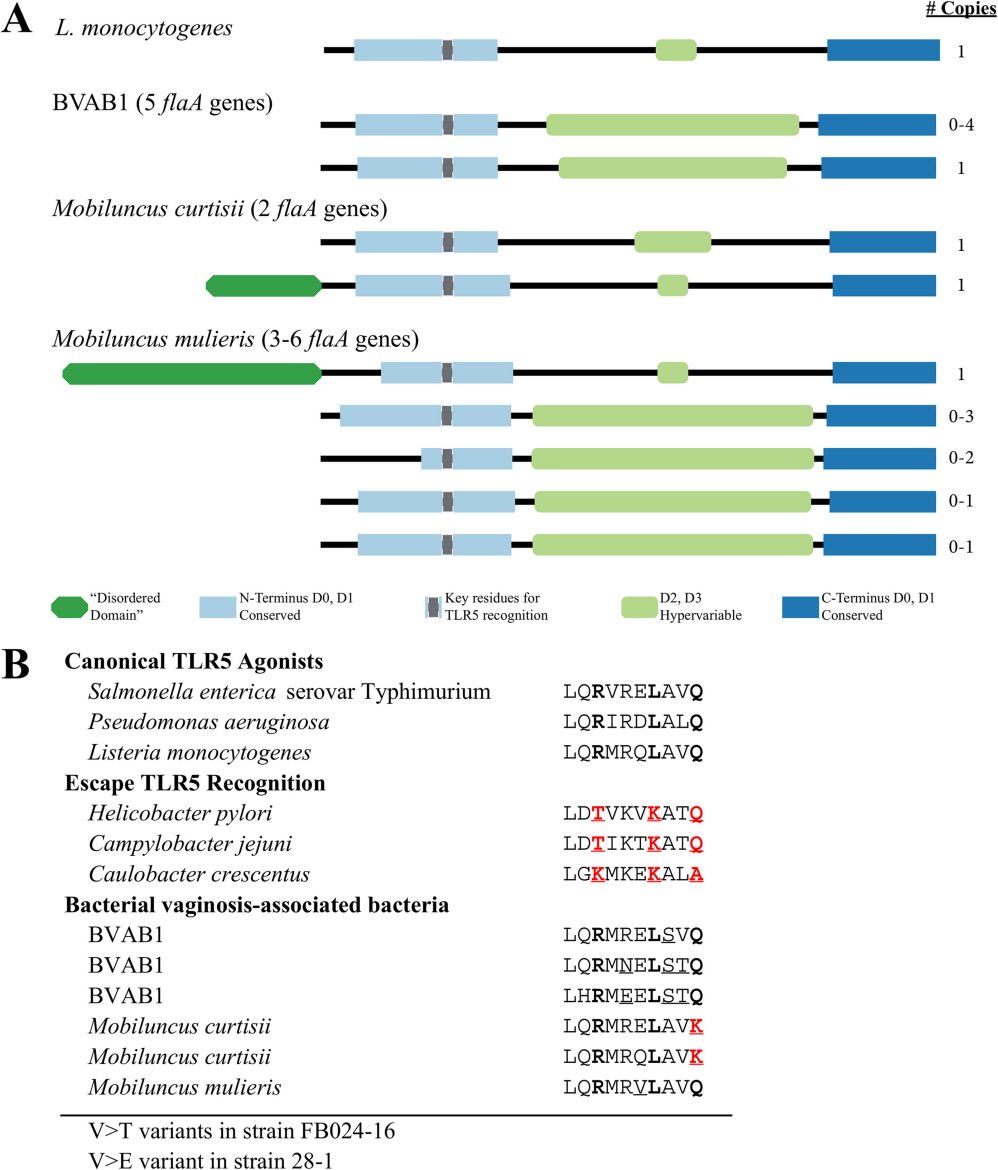
Alignment of FlaA amino acid sequences reveals diversity among BVAB. (A) Schematic depicting alignments of FlaA amino acid sequences from BVAB1 (1 MAG, 1 vaginal metagenome), *M. curtisii* (3 genomes), and *M. mulieris* (4 genomes). Hypervariable domains are in green; blue represents conserved regions. The width of each box is proportional to the number of amino acids in each domain. Gray boxes highlight the location of the 10-amino-acid sequence within N-terminal D0/D1 previously found to be highly conserved and necessary for TLR5 recognition (24). These alignments revealed a novel disordered domain attached to the N terminus of *M. curtisii* (112 amino acids [aa]) and *M. mulieris* (252 aa) FlaA sequences. This FlaA sequence with the disordered domain was present in each of the *M. mulieris* and *M. curtisii* genomes analyzed. While the C-terminal D0 and D1 domains were relatively conserved across species, size of D2 and D3 hypervariable region ranged from 29 to 273 aa. N-terminal D0 and D1 domains ranged from 88 to 150 aa. (B) Details of the 10-amino-acid sequence recognized by TLR5, with key amino acid residues in bold. Underlined residues are departures from the sequence in canonical TLR5 agonists; residues in red are mutations found to be important for both TLR5 recognition and flagellar motility. FlaA from BVAB1 and *M. mulieris* have no mutations in key residues and are anticipated to act as TLR5 agonists. The Q-to-K mutation in the 10th amino acid in *M. curtisii* may result in decreased TLR5 agonism.

The data presented thus far led us to hypothesize that TLR5 deficiency protects against colonization with BVAB1 and *M. mulieris* (but not *M. curtisii*). To explore how such a relationship could be mediated, we investigated the flagellin amino acid sequences (FlaA) known to be encoded by these species, though the FlaA sequences from the women in our study were not specifically assessed. We examined FlaA amino acid sequence alignments for the appearance of conserved and variable domains. These sequences were compared to characterized TLR5 agonists (24) and sequences from flagellated bacteria that are known to escape TLR5 recognition (13). We noted differences in the number of *flaA* genes present in each organism. *M. curtisii* isolates consistently possessed two *flaA* genes each, while *M. mulieris* genomes contained between three (strain ATCC 35239) and six (strains FB024-16 and 28-1) *flaA* genes, each of which appeared sequentially distinct, suggesting that the observations noted are not a result of sequencing artifacts or errors. In comparison, most Listeria monocytogenes isolates possess a single *flaA* gene. As depicted in Fig. 3A, FlaA from *Mobiluncus* spp. were highly variable. Both *M. curtisii* and *M. mulieris* encode a potentially novel FlaA sequence with a so-called disordered domain attached to the highly conserved N-terminal. This disordered domain was approximately 112 amino acids long in *M. curtisii* and 252 amino acids long in *M. mulieris*. The hypervariable D2 and D3 regions are bound by antibodies to the flagellar filament; among *Mobiluncus* species, these regions varied in length as well. Structural threading using phyre2 (25) revealed no structural homologies between the disordered domains contained in *M. curtisii* or *M. mulieris*, although the C termini of these genes mapped with 100% confidence to well-described flagellin crystal structures.

The BVAB1 MAG appeared to contain one full-length *flaA* gene in addition to three *flaA* gene fragments with significantly shortened N and C termini, possibly suggesting that this organism likewise possesses multiple *flaA* genes. We further examined the presence of *flaA* genes in a metagenome assembly we generated from a vaginal sample in which BVAB1 was the dominant microorganism (∼70% relative abundance). In this assembly of over 4,000 contigs ranging from 0.5 to 343 kb in length, we identified five full-length *flaA* genes on long, high-coverage contigs (39 to 189 kb in length; >40× coverage), all of which aligned with 97 to 100% amino acid identity to the FlaA sequences present in the BVAB1 MAG (9).

Using the four high-coverage full-length FlaA sequences from our BVAB1 assembly, the BVAB1 MAG FlaA sequence, and those from published *Mobiluncus* genomes, we analyzed a 10-amino-acid sequence in the N-terminal conserved domain that is highly conserved across flagellated species and predicted to bind TLR5 (24). Within this 10-amino-acid sequence, we examined three key amino acids, shown by Smith et al. (12) to be important for both TLR5 recognition and flagellar motility. Based on our epidemiological findings, we predicted that BVAB1 and *M. mulieris* would have flagellin amino acid sequences with key recognition and motility residues conserved, while *M. curtisii* would have at least one key amino acid substitution.

Consistent with our hypothesis, the three key amino acids we examined were conserved in both isoforms of BVAB1 FlaA as well as all *M. mulieris* isolates (Fig. 3B). Thus, we predicted that BVAB1 and *M. mulieris* would stimulate a TLR5 response. *M. curtisii* isolates consistently had a single glutamine-to-lysine (Q-K) substitution in the last amino acid of the TLR5 recognition sequence (Fig. 3B). However, most species that escape TLR5 recognition possess substitutions in 2 or 3 of 3 key residues; thus, we predicted incomplete abrogation of TLR5 agonism resulting from stimulation with *M. curtisii* FlaA.

### Partially purified flagellin from *Mobiluncus* spp. stimulates a TLR5-dependent immune response.

**FIG 4:**
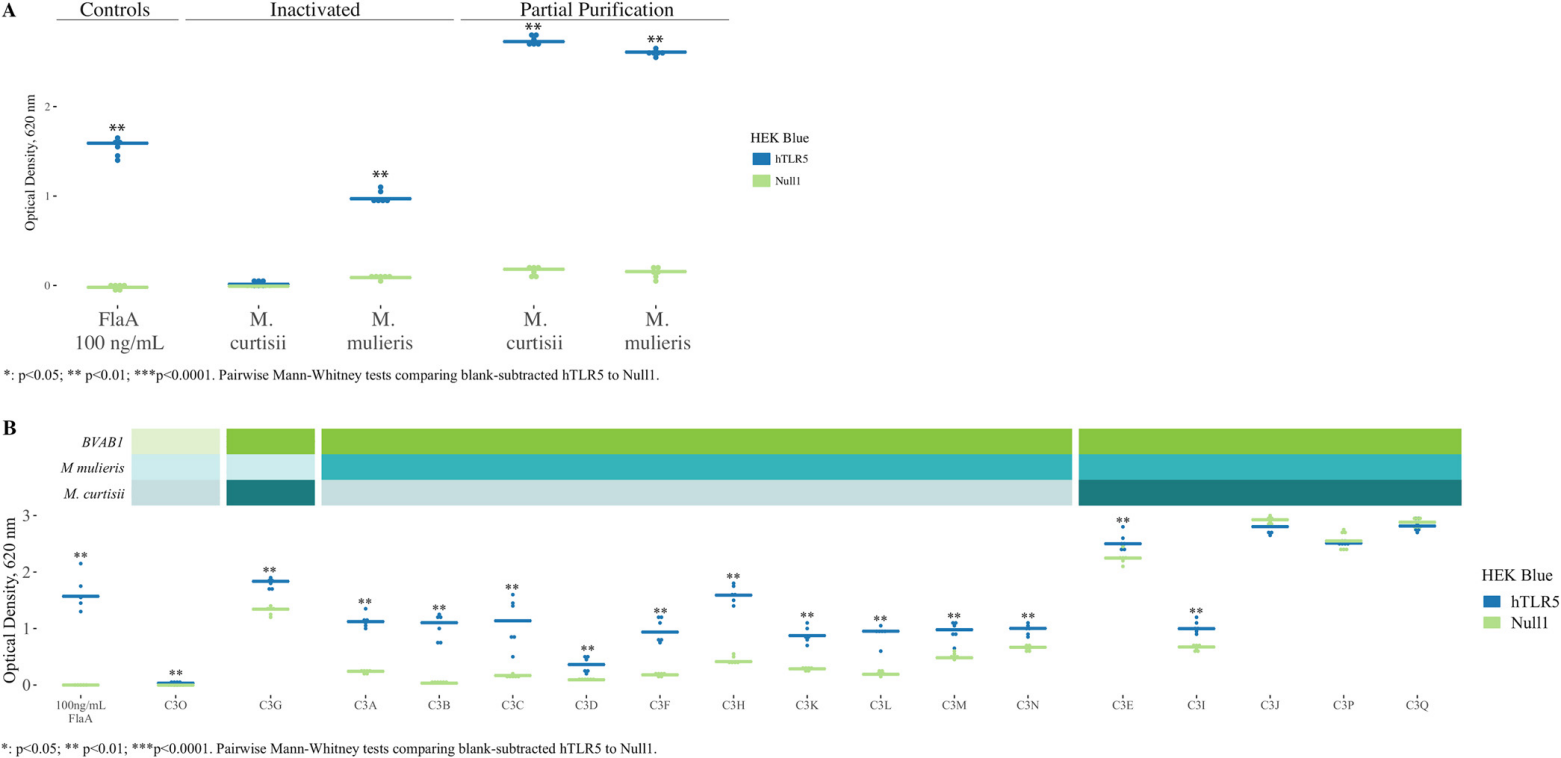
TLR5 responses associated with heat-inactivated *M. mulieris*, partially purified FlaA from *M. curtisii*, and vaginal fluid from women colonized with BVAB1. HEK293 cells transfected with a plasmid containing a NF-κB-driven secretory embryonic alkaline phosphatase (SEAP) reporter alone (HEK Blue Null1) or in addition to a plasmid encoding hTLR5 (HEK Blue hTLR5) were acquired from Invivogen (San Diego, CA). Cells were stimulated with heat-inactivated *Mobiluncus* (A, left), partially purified flagellin from *Mobiluncus* (A, right), or cervicovaginal lavage (CVL) fluid from validation cohort study participants with various concentrations of BVAB1, *M. mulieris*, and *M. curtisii* (B). OD_620_ indicates SEAP activity as a readout of NF-κB inflammatory response. Each biological replicate consisted of three technical replicates. (A) Heat-inactivated *M. mulieris* stimulates a TLR5-specific inflammatory response, while heat-inactivated *M. curtisii* does not stimulate TLR5. However, partially purified flagellin monomers from *M. curtisii* stimulate a TLR5-dependent immune response. As expected, partially purified flagellin from *M. mulieris* also stimulates a TLR5-dependent immune response. The positive control was flagellin from Bacillus subtilis (FlaA); the negative control was PBS. Results depict blank-subtracted values from two biological replicates. (B) Vaginal fluid from cohort 3 participants extensively colonized with BVAB1 stimulate TLR5-dependent inflammatory responses. Cervicovaginal lavage (CVL) fluid from women extensively colonized with BVAB1 (>10^8^ 16S rRNA gene copies per swab)—in the absence of substantial colonization with *M. curtisii*—stimulates a TLR5-mediated inflammatory response. In the presence of *M. curtisii*, a non-TLR5-specific response is stimulated. Results depict blank-subtracted values from two biological replicates. On the *x* axis, the designations C30, C3G, etc., correspond to randomly assigned labels given to samples collected from cohort 3 (C3) and map to Table S3. Bright color bands at the top indicate that 16S rRNA gene copies in the vaginal fluid were greater than the following thresholds for the bacterial species indicated to the left of the bar, while dim color bands indicate that they were less than the threshold: green = 10^8^ BVAB1; blue = 500 *M. mulieris*; aqua = 10^5^ *M. curtisii*.

We tested our sequence-based predictions with a HEK TLR5 reporter cell line stimulated with heat-inactivated *M. curtisii* ATCC 35241 and *M. mulieris* UPII 28-I. Even though HEK cells were inoculated with the equivalent of approximately 10^6^ CFU of *M. curtisii* (multiplicity of infection [MOI] = 79) or 10^3^ CFU of *M. mulieris* (MOI < 1), we consistently found that *M. mulieris* produced a TLR5-specific NF-κB inflammatory response, while *M. curtisii* did not (*P* = 0.002) (Fig. 4A).

Because the presence of other bacterial products may inhibit agonism of TLR5 by *M. curtisii*, we partially purified FlaA using methods adapted from the work of Smith et al. (12). Confirming results obtained using whole, heat-inactivated bacteria, partially purified flagellin from *M. mulieris* stimulated a TLR5 immune response. In contrast to whole, heat-killed *M. curtisii* organisms, stimulation with partially purified flagellin from *M. curtisii* surprisingly elicited a TLR5-dependent immune response (Fig. 4A). This contrast in TLR5 stimulation resulting from *M. curtisii* heat-inactivated whole cells versus partially purified flagellin monomers is unexpected and is explored in greater detail in Discussion.

### Cervicovaginal lavage fluid from women colonized with BVAB1 stimulates a TLR5-dependent immune response.

Unlike *Mobiluncus* species, BVAB1 has not (to our knowledge) been grown in pure culture. Thus, we obtained cervicovaginal lavage (CVL) fluid supernatant from women enrolled in a third cohort (cohort 3) colonized to various degrees with *M. curtisii* and *M. mulieris* and extensively colonized with BVAB1 (Table S3). HEK Blue Null1 and hTLR5 cells were incubated with CVL fluid supernatant and examined for NF-κB activation. CVL fluid from women extensively colonized with BVAB1 and *M. mulieris*—with fewer than 10^5^ *M. curtisii* 16S rRNA gene copies per swab—stimulated a TLR5-mediated NF-κB response. When more than 10^5^ *M. curtisii* 16S rRNA gene copies per swab were present, an NF-κB response was stimulated, but this response did not appear to be mediated through TLR5 specifically, as evidenced by inflammatory response generated in Null1 cells (Fig. 4B). Results shown are the aggregate of two biological replicates.

### Vaginal IL-8 concentration does not correlate with absolute concentration of BVAB1, *M. mulieris*, or *M. curtisii* in TLR5-sufficient women.

As a corollary to our HEK reporter cell assay results, we hypothesized that proinflammatory chemokine IL-8 concentrations would correlate with concentrations of BVAB1, *M. mulieris*, and *M. curtisii* in TLR5-sufficient women. To test this prediction, we assayed IL-8 concentrations in vaginal swab supernatant from the TLR5-sufficient validation cohort participants using enzyme-linked immunosorbent assay (ELISA). However, we found no significant correlation between vaginal concentrations of IL-8 and flagellated BVAB (data not shown). Similarly, no correlation was observed between vaginal concentrations of IL-8 and Gardnerella vaginalis (Fig. S4).

### Absolute concentrations of BVAB1 and *M. mulieris* are correlated.

The absence of an association between IL-8 and flagellated bacterial concentrations led us to hypothesize that these bacteria may evade the immune response. Specifically, we hypothesized that the FlaA containing a disordered N-terminal domain encoded by *M. curtisii* and *M. mulieris* may act as a TLR5 antagonist. BVAB1 and *Mobiluncus* spp. often co-occur (6, 26); because BVAB1 does not encode a flagellin with a disordered domain, BVAB1 may benefit from the presence of *Mobiluncus* spp. if these bacteria produce a factor that mediates immune evasion.

**FIG 5:**
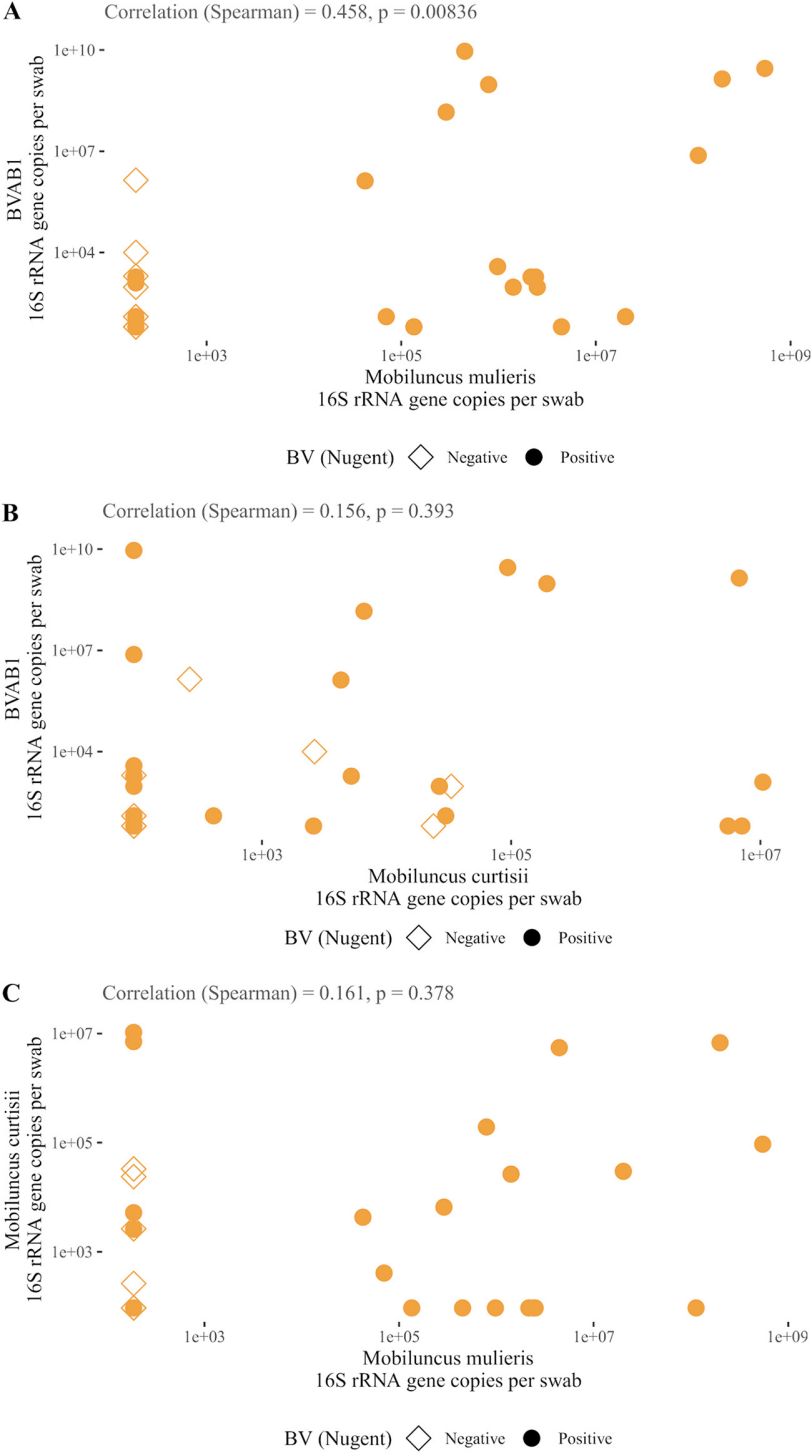
Correlations between flagellated BVAB concentrations among TLR5-sufficient women. Among TLR5-sufficient women in the validation cohort, there was a statistically significant, positive correlation between BVAB1 and *M. mulieris* colonization (A); however, BVAB1 and *M. curtisii* were not correlated (B), nor were *M. curtisii* and *M. mulieris* (C).

Based on this premise, we hypothesized that BVAB1 concentrations would correlate with both *M. curtisii* and *M. mulieris* concentrations. To test this hypothesis, we examined pairwise correlations between the three flagellated species in our validation cohort. We found a significant, positive correlation between concentrations of BVAB1 and *M. mulieris* (Fig. 5A) (Spearman correlation = 0.46; *P* = 0.008). There were no significant correlations between BVAB1 and *M. curtisii* (Fig. 5B) or between *M. mulieris* and *M. curtisii* (Fig. 5C). Together, these data suggest that BVAB1 and *M. mulieris* may benefit from TLR5 stimulation combined with host inflammatory attenuation or dysregulation *in vivo*.

## DISCUSSION

Although it has been over 3 decades since flagellated bacteria were observed in vaginal fluid from women with BV (3, 4, 11), the role of TLR5-modulated innate immune recognition of flagellin has never been explored in this syndrome. Here, we demonstrated an epidemiological association between genetically encoded TLR5 deficiency and decreased risk of Nugent scores of 9 or 10 (associated with the presence of *Mobiluncus* morphotypes on Gram stains) in the discovery cohort (2). While these results were not replicated in our validation cohort, our validation cohort demonstrated a trend consistent with discovery cohort findings that may be limited by sample size and power. Exploring how TLR5 deficiency affects colonization, we found decreased absolute abundance of BVAB1 by quantitative PCR in women with TLR5 deficiency in both discovery and validation cohorts. In the validation cohort, we found decreased absolute abundance of *M. mulieris* in women with TLR5 deficiency without any differences in *M. curtisii* absolute abundance detected. We explored the mechanisms underlying this surprising relationship first by exploring FlaA amino acid sequences encoded by these flagellated BVAB. We found that while FlaA proteins encoded by BVAB1 appear very similar to well-characterized TLR5 agonists, FlaA proteins encoded by *Mobiluncus* spp. are highly variable, including a FlaA predicted to have a large, N-terminal disordered domain not found on other flagellins. We demonstrated robust TLR5 agonism resulting from stimulation with partially purified FlaA from *Mobiluncus* spp., although *M. curtisii* heat-inactivated cells did not stimulate a TLR5 response. One limitation of our approach was that extracts of flagellin were crude, and it is possible that other proteins were present that impacted the TLR5 NF-κB inflammatory response *in vitro* (Fig. 4B). Because BVAB1 has not been grown in pure culture, we used CVL fluid supernatants from women colonized with various concentrations of BVAB1 and found that CVL fluid from women extensively colonized with BVAB1 stimulates a TLR5-dependent NF-κB inflammatory response. These results suggest decreased accessibility of the portion of flagellin that interacts with TLR5 in whole *M. curtisii* cells. Last, we explored IL-8 concentration in TLR5-sufficient women and found no correlation between concentrations of TLR5-agonist species (BVAB1, *M. mulieris*, and *M. curtisii*) and IL-8. However, absolute concentration of BVAB1 and *M. mulieris* were correlated in TLR5 sufficient women, suggesting possible growth synergy between these two organisms, such as by cross-feeding of nutrients.

Although TLR5 expression has been noted in multiple cell types in the female reproductive tract, including epithelial cells in the vagina and cervix (27), it is unclear whether TLR5 is primarily present on the epithelial surface, where it can interact with the resident microbiota, or on the basolateral surface of the epithelium, where it might detect invading pathogens. Ali et al., in their study of recurrent urinary tract infection in women (28), demonstrated that both the immortalized vaginal cell line VK2 E6/E7 and primary vaginal epithelial cell lines respond to flagellated Escherichia coli via TLR5/NF-κB with production of human beta-defensin-2 protein, highlighting the important role of TLR5 signaling in this cell type and the potential of secreted effectors to impact the microbiota. Another limitation of our *in vitro* work is that HEK293 cells are of renal origin, and vaginal epithelial cells may have different responses to flagellated bacteria.

We put forth the following hypotheses based on the data presented here. We hypothesize that *M. mulieris* and BVAB1 require a host inflammation-associated product of TLR5 agonism in order to robustly colonize. We have shown that *M. mulieris* and BVAB1 stimulate TLR5 and provoke a NF-κB-dependent proinflammatory response; we predict this creates a niche for these species to occupy. This inflammatory response may decrease availability of host nutrients used by vaginal lactobacilli (e.g., glycogen or metal ions, such as Mn^2+^ and Mg^2+^) or cause the direct elimination of lactobacilli (via release of host antimicrobial proteins, for example). *Mobiluncus* spp. have been found by electron microscopy to localize in close association with host epithelial cells (3), suggesting that these flagellated bacteria are able to attach to host cells. The innate immune response to flagellin could facilitate attachment sites used by BVAB1 or *M. mulieris*—this could occur in the form of upregulation or posttranslational modifications to host cell surface proteins. Alternatively, considering the reproductive imperative to prevent infection of the uterus, TLR5 agonism could lead to changes in mucin production in the cervix to reinforce a mechanical barrier to invasion; mucin could be a nutrient source for these bacteria. Finally, the lack of IL-8 response to flagellated BVAB in TLR5-sufficient women may indicate that flagellated BVAB themselves modulate the immune response or that flagellated BVAB benefit from other microbes present in BV that secrete substances with immunomodulatory effects (e.g., short-chain fatty acids and proteases).

While the TLR5-mediated immune response to flagellated bacteria possibly supports robust vaginal colonization with some species, we hypothesize that this response results in protection from ascending infection with these bacteria. This question could be examined in cohort studies of pelvic inflammatory disease or preterm birth. In addition, future studies could take a multi-omics approach to examine how flagellin stimulation of lower-genital-tract mucosa changes gene expression, as well as the proteome, glycome, and metabolome. Last, the innate immune response to flagellated organisms is mediated by TLR5, which recognizes extracellular flagellin, and NLRC4, which recognizes cytosolic flagellin (29). Here, we focused on TLR5, as NLRC4 does not have any well-characterized, common SNPs. As data on NLRC4 genetic variation emerges, future studies could examine whether NLRC4 plays a role in vaginal colonization with flagellated organisms.

The disordered domain present at the N termini of some *M. mulieris* and *M. curtisii* flagellin alleles may constitute a novel bacterial adaptation for influencing host immune response. These disordered domains have highly variable sequences between species, which leads us to hypothesize that these domains play unique roles in the life cycle of each species. Intrinsically disordered protein regions, or domains, are common in nature and frequently confer complementary functions to ordered domains in a protein, such as an active site (30). These domains can affect protein structure and function through many mechanisms, such as by posttranslational modification, folding, creation of “fuzzy complexes,” and interactions with other proteins (31). Disordered protein regions have been described in genes encoding flagellin and affect function of the flagellar motor (32, 33). We hypothesize that *M. mulieris* uses this disordered domain for steric inhibition of TLR5 dimerization and downstream immune response. Moreover, these disordered-domain-containing *flaA* genes may be differentially expressed depending on stage of colonization. For example, because this bulky disordered domain may interfere with stacking of flagellin monomers (and thus would inhibit motility), this particular *flaA* gene may be expressed only after the bacterium has attached to a host cell. Additional studies are necessary to establish whether these novel *flaA* genes are expressed and to identify sigma factors associated with upregulation of these novel *flaA* genes to elucidate the conditions under which these genes are expressed.

Conversely, we believe that disordered domain flagellin genes may have a different function in *M. curtisii*. We hypothesize that the disordered domain provides greater stability to the flagellar filament, shielding the conserved regions of FlaA from TLR5 and resulting in immune evasion. We hypothesize that this disordered-domain-mediated stability is the mechanism underlying the lack of TLR5 stimulation from heat-inactivated *M. curtisii* (where the domain is intact) versus the robust TLR5 stimulation resulting from partially purified *M. curtisii* flagellin monomers that we describe here.

Our data suggest that the condition defined clinically and microbiologically as BV is not caused by the presence of *flaA* in BVAB1 or *M. mulieris* and its interaction with TLR5. Muzny et al. demonstrated no significant difference in BVAB1 colonization in women who develop incident BV compared to those who do not in a cohort of African-American women who have sex with women (34). Although the data presented in our work suggest the possibility that vaginal colonization with BVAB1 and *M. mulieris* may be facilitated by signaling of TLR5 through a functional ligand of FlaA, we have no evidence that BVAB1 or its FlaA gene plays a role in incident BV. Nonetheless, data presented here suggest that vaginal colonization with BVAB1 and *M. mulieris* may be facilitated by signaling of TLR5 through a functional ligand of FlaA.

Because TLR5 deficiency appears to protect against colonization with specific BVAB but does not prevent the appearance of clinically or microbiologically defined BV, it is unlikely that treatment with TLR5 antagonists would be an effective preventive measure against BV. However, it is possible that TLR5 antagonism could protect against BV caused by BVAB1 dominant communities. The data presented here suggest that BV involving BVAB1 and/or *M. mulieris* may have a distinct inflammatory phenotype differentiating it from BV not involving these species. These observations open new avenues for understanding how specific vaginal bacteria interact with the host to produce adverse health outcomes in women.

## MATERIALS AND METHODS

### Cohort enrollment.

Two independent cohorts were enrolled: a longitudinal discovery cohort of 213 women and a cross-sectional validation cohort of 111 women. Study participants gave written, informed consent to participate in both studies and were enrolled from the Public Health Seattle and King County Sexually Transmitted Diseases Clinic (PHSKC STD Clinic) and the University of Washington Infectious Diseases/Virology Research Clinic. Each study enrolled both women with and without prevalent BV, and cohorts were composed of nonpregnant women of reproductive age.

The discovery cohort (cohort 1; NIH project R01 AI061628) participants were enrolled between 2012 and 2015 and had regularly scheduled follow-up study clinic visits (at 30, 60, 90, and 180 days and approximately 1 year after enrollment). This study was approved by the Institutional Review Board (IRB) at the Fred Hutchinson Cancer Research Center (number 7683). Written, informed consent was obtained from all study participants. Study clinicians collected behavioral, medical, and sexual history, performed speculum exams, collected vaginal swabs; and assessed women for BV using Amsel’s criteria. Swabs collected at these visits were used for Gram staining and Nugent scoring for microbiological diagnosis of BV. Cohort 1 participants self-collected vaginal swabs on a daily basis (as previously described [35]). These swabs were collected daily for up to 90 days and then weekly for an additional 90 days. Participants mailed these swabs to our laboratory on a daily basis, or alternatively were allowed to store them in their home freezer for batch transportation to their study clinic appointments. Upon arrival at our laboratory, samples were stored at −80°C until thawed for DNA extraction and downstream analysis.

A subset of 75 participants from cohort 1 were selected for characterization of the vaginal microbiome via genus- and species-specific quantitative PCR. Participants were selected based on the proportion of daily samples collected and prevalent or incident BV during enrollment. All participants selected for microbiome characterization collected at least 30 consecutive daily samples. Sixty-one of these participants developed at least one episode of BV by Nugent score during their enrollment.

The validation cohort (cohort 2; NIH project R56 AI052228-06A2) participants were enrolled between 2012 and 2013. Enrollment of cohort 2 was approved by the Fred Hutchinson Cancer Research Center IRB (number 1789). Written, informed consent was obtained from all study participants. These women were assessed for BV at enrollment using procedures similar to those described for cohort 1. Swabs collected at enrollment were stored at −80°C, transported to the laboratory on dry ice, and stored at −80°C.

In addition to the discovery (cohort 1) and validation (cohort 2) cohorts used for genetic analyses, a third clinical cohort (cohort 3; NIH project R01 HG005816) was enrolled between April 2011 and July 2013 from the PHSKC STD Clinic. Cohort 3 was approved by the Fred Hutchinson Cancer Research Center IRB (number 7363). Written, informed consent was obtained from all study participants. In cohort 3, study clinicians collected cervicovaginal lavage (CVL) fluid by inserting 5 ml sterile saline and then using a pipette to transfer the saline into a 15-ml conical tube. The resultant fluid was divided into two 15-ml conical tubes and centrifuged for 10 min at 800 × *g*. The supernatant was separated into 2 cryovial tubes and frozen at −80°C. After arrival in the lab from the clinic, CVL fluid supernatants were thawed, spun down at 1,000 × *g* for 20 min (to remove any residual human cells), and aliquoted. Single aliquots were thawed for downstream experiments. These CVL fluid specimens from cohort 3 were used to assess cytokine concentrations and TLR5 agonism.

### Single nucleotide polymorphism genotyping.

TLR5 rs5744168 was genotyped using a commercially available TaqMan assay (Thermo Fisher, Waltham, MA). The discovery cohort (cohort 1) assay was run on a Fluidigm 48.48 chip. The validation cohort (cohort 2) assay was run on a Fluidigm 96.96 chip. Genotypes that could not be resolved on those chips were run as single SNP assays using StepOnePlus (Applied Biosystems, Foster City, CA). A dominant model was chosen for analysis based on published data (18).

### DNA extraction and quantitative PCR.

DNA was extracted using commercially available bacterial DNA extraction kits, and quantitative PCR (qPCR) was performed as described elsewhere (1, 36).

In cohort 1, BVAB1 was chosen for quantitation based on its possession of a putative flagellin gene. Gardnerella vaginalis and Lactobacillus crispatus were selected as representative bacteria present in BV and in its absence, respectively.

Cohort 2 was assayed for the same species and genera as cohort 1 with the addition of Mobiluncus mulieris and Mobiluncus curtisii. To quantify the latter two species, a multiplex qPCR assay was developed specifically for this study, using forward primer Mobi1007F (5′-CTTACCAAGGCTTGACATACA-3′), reverse primer Mobi1088R (5′-ACCACCTGTACACCACC-3′), and the species-specific probes Mmuli1034_1054 (5′-VIC-CATGCCAGAGATGGTGTGG-MGB-NFQ-3′) and Mcurt1034_1054 (5′-FAM-TGGTTCCAGAGATGGGCCAG-MGB-NFQ-3). Precycling conditions were 50°C for 2 min followed by 95°C for 20 s and then 45 cycles of 95°C for 2 s, 57°C for 20 s, and 72°C for 20 s.

### Amino acid sequence analysis of flagellin from BVAB1, *M. curtisii*, and *M. mulieris*.

To obtain full-length BVAB1 FlaA sequences, we consulted a BVAB1-dominant (∼70% relative abundance) vaginal metagenome generated from a CVL sample collected from a participant in another study of women of reproductive age at the PHSKC STD Clinic (cohort 4; recruited September 2006 to June 2010; NIH R01 AI061628 [1, 6]). DNA was extracted from the CVL pellet as described above and used to generate a TruSeq library for sequencing on the HiSeq 2500 instrument (Fred Hutchinson Cancer Research Center Genomics Shared Resource). Raw reads were evaluated for quality using FastQC (http://www.bioinformatics.babraham.ac.uk/projects/fastqc/), and reads aligning to the hg19 reference were removed with Best Match Tagger (ftp://ftp.ncbi.nlm.nih.gov/pub/agarwala/bmtagger/). Deduplication was then performed using Picard’s EstimateLibraryComplexity (http://broadinstitute.github.io/picard/), followed by end trimming with trimBWAstyle.usingBam6.pl (-o 33 -q 3) (https://github.com/SycuroLab/delaCruz_flaA_TLR5). Pairs with one or both reads shorter than 60 nucleotides (nt) were discarded, yielding 3,351,401 clean nonhuman read pairs (100 bp). These were assembled using SPAdes version 3.5.0 with the following parameters: -k 21,33,55,77 –phred-offset 33 (36). The resulting assembly was filtered to remove contigs less than 500 nt, leaving 4,036 contigs (*N*_50_ = 19,073 nt). Contigs were annotated using the classic RAST pipeline (37, 38) and aligned to the BVAB1 MAG FlaA sequences using BLASTP (39).

*M. mulieris* (35239, 35243, 28-1, FB024-16, and FDAARGOS_303) and *M. curtisii* (35242, 43063, and 51333) amino acid sequences annotated as “FlaA” were obtained from the PATRIC database (37); in addition, FliC sequences from Pseudomonas aeruginosa and Salmonella enterica serovar Typhimurium and FlaA sequences from Listeria monocytogenes were obtained. Sequences were aligned using the MUSCLE algorithm implemented in Geneious (38). After further inspection, *M. mulieris* FDAARGOS_303 was excluded from the analysis, as it contains 16 sequences annotated as FlaA in its genome and was thus an outlier among the other *M. mulieris* whole-genome sequences. The amino acid sequences aligning to the previously described 10-amino-acid TLR5 recognition sequences (24) present in well-characterized agonists (P. aeruginosa, S. enterica serovar Typhimurium, and L. monocytogenes) were identified as the probable recognition sequences of BVAB1 and *Mobiluncus* spp. The gross alignments were compared to assess sequence length and the presence of conserved amino acid domains D0, D1, D2, and D3 (reviewed in reference 39). Additional structural threading was performed using phyre2 (25).

### Preparation of heat-inactivated *M. curtisii* and *M. mulieris*.

*M. curtisii* ATCC 35241 and *M. mulieris* UPII 28-I were first grown on *Brucella* agar with 5% sheep blood (supplemented with hemin and vitamin K; Hardy Diagnostics, Santa Maria, CA) and were passaged twice before use in experiments to ensure purity. We verified motility of these strains by observing movement of cells grown in broth under wet mount and growth in motility agar (0.25% agar) (5).

A loop of bacterial cells scraped from the plate was suspended in fresh PYGmod broth and used to inoculate disposable culture tubes containing 5 ml PYGmod (40). Bacteria were incubated for 72 h at 37°C (without shaking) in an anaerobe chamber (Anaerobe Systems, Morgan Hill, CA), after which cells were pelleted, washed 3 times, resuspended in 3 ml PBS, and heat treated at 65°C for 30 min. Heat-inactivated cells were aliquoted and stored at −20°C until use. Each biological replicate of stimulation experiments using these heat-inactivated cells was performed using a different aliquot to avoid multiple freeze-thaw cycles.

### Partial purification of flagellin from *Mobiluncus* spp.

We partially purified flagellin using a protocol adapted from the work of Smith et al. (12). Briefly, 400 ml Columbia Broth (catalog no. DF0944170; Difco/Fisher Scientific) with 5% horse serum (catalog no. H1138; Sigma-Aldrich) was inoculated with *M. mulieris* or *M. curtisii* and grown anaerobically, without shaking. After 3 days incubation at 37°C, bacteria were pelleted at 6,400 × *g* for 10 min, resuspended in 60 ml sterile phosphate-buffered saline (PBS), and placed in a blender (Hamilton Beach). To shear flagellar filaments from cells, bacteria were blended at high speed for 3 min and then centrifuged at 6,400 × *g* for 15 min to pellet bacterial cells. Supernatant was removed and centrifuged at 100,000 × *g* for 1 h at 4°C to pellet flagellar filaments. The resulting supernatant was then removed, and the pellet was resuspended in 1 ml sterile PBS and centrifuged again at 100,000 × *g* for 1 h at 4°C. After removal of the supernatant, filaments were resuspended in 1 ml sterile PBS and heat treated in a 70°C water bath for 20 min to dissociate filaments into monomers. Monomers were separated from undissociated filaments through centrifugation (100,000 × *g*, 1 h, 4°C). Supernatant was transferred to a separate microcentrifuge tube, stored at 4°C, and used for TLR5 stimulation assays within 1 week.

### Identification of bacteria and cervicovaginal lavage fluid capable of TLR5 agonism.

HEK Blue Null1 (Invivogen, San Diego, CA; catalog no. hkb-null1, lot no. 39-01-hkbnull1) and HEK Blue hTLR5 (Invivogen, San Diego, CA; catalog no. hkb-htlr5, lot no. 38-01-hkbhtlr5) cells were grown according to the manufacturer’s protocol. These cell lines were derived from HEK293 cells and were each transfected with a plasmid encoding an NF-κB-driven secretory embryonic alkaline phosphatase (SEAP) reporter. Per the manufacturer’s package insert, HEK Blue hTLR5 expression is 20 to 100 times the endogenous hTLR5 expression in HEK293 cells.

For each biological replicate, cells were thawed from frozen stocks (passage number 3 to 5), washed in fresh Dulbecco’s modified Eagle medium (DMEM) (Gibco, Thermo Fisher, Waltham, MA), and plated on 65-mm tissue culture-treated dishes in 5 to 6 ml medium containing selective antibiotics. The following day, cells were passaged to 150-mm dishes and incubated with selective antibiotics until 50 to 60% confluent. Medium was replaced every day to ensure effective concentrations of selective antibiotics. Before use in an experiment, cells were washed with fresh PBS, then scraped in 2 to 3 ml PBS, resuspended, and counted. Concentrated cells in PBS were resuspended in fresh HEK-Blue detection medium (InvivoGen, San Diego, CA; catalog no. hb-det2) at an approximate concentration of 140,000 cells/ml. Each plate included purified FlaA from Bacillus subtilis (InvivoGen, San Diego, CA; catalog no. tlrl-pbsfla) as a positive control and PBS (Gibco/Thermo Fisher, Waltham, MA) as a negative control. Each experiment included three technical replicates per condition. Optical density at 620 nm (OD_620_) was measured using a VersaMax microplate reader (Molecular Devices, San Jose, CA).

As noted in the manufacturer’s product notes, any exogenous source of NF-κB activation will produce a SEAP-response in both HEK Blue Null1 and HEK Blue hTLR5 cells. These include cytokines such as tumor necrosis factor alpha (TNF-α) and ligands for any endogenously expressed pattern recognition receptors (TLR3, TLR5, and NOD1).

### Measurement of IL-8 in vaginal fluid.

Vaginal swabs were placed in 2-ml microcentrifuge tubes and vortexed with 450 µl sterile saline for 1 min at high speed. Swabs were removed from the tube and discarded. The cellular material in the tube was pelleted by centrifuging at 14,000 rpm for 10 min at 4°C. Supernatant was then separated from the cell pellet and stored at −80°C.

IL-8 concentration in swab supernatants was determined by ELISA (R&D Systems, Minneapolis, MN; catalog no. DY208). Samples were warmed to room temperature before being assayed. Any samples that were undetectable were imputed at half the level of detection.

### Statistical analysis.

Data analysis was performed using R version 3.6.0 for Windows.

### (i) TLR5 deficiency and bacterial vaginosis.

Analyses were restricted to women with resolved TLR5 genotypes. Because recent diagnosis of BV is a strong predictor of recurrent BV, we estimated the effect of TLR5 deficiency on risk of incident BV in the discovery cohort by restricting our analysis to women with BV at enrollment in the discovery cohort. In this subcohort, we used Cox proportional hazards regression (R package “survival,” version 2.41.3) to estimate differences in time to first diagnosis of BV between TLR5-sufficient and -deficient women. In the validation cohort, logistic regression was used to estimate cross-sectional differences in odds of clinically and microbiologically defined BV.

### (ii) Bacterial colonization in the discovery cohort.

To assess differences in bacterial colonization in the discovery cohort, we used generalized linear regression with random effects at the study participant level to control for repeated measurements. This analysis was limited to 54 (of 214 total) study participants who (i) had qPCR data available; (ii) had prevalent BV at enrollment or developed incident BV during the study; (iii) had bacterial colonization data for least 7 days immediately following an episode of BV, and (iv) were not missing key demographic data (age, race, history of BV, or use of hormonal contraception). The analysis was further restricted to participants with resolved TLR5 genotypes. A schematic representation of how enrollees were included or excluded from the analysis can be found in Fig. S5.

This subset included 16 TLR5-deficient participants, and we selected TLR5-sufficient participants in a 2:1 ratio using MatchIt (v3.0.2) to balance risk factors known to influence risk of BV (age, race, hormonal contraception, and sex with men/women). To be included in the analysis, participants were required to have no missing data used for matching; no imputation was performed for this analysis. Comparison of risk of recurrent BV, longitudinal percentage with microbiological diagnosis of BV, and demographic characteristics used for matching are available in Fig. S1.

### (iii) Bacterial colonization in the validation cohort.

In the validation cohort, bacterial colonization data were generated for all study participants. We began with 8 TLR5-deficient study participants and matched these in a 4:1 ratio to TLR5-sufficient participants with similar risk of BV (age, race, hormonal contraception, and douching) using MatchIt (v3.0.2). Analysis was limited to participants with resolved TLR5 genotypes who were not missing any demographic data used for matching; no imputation was performed for the analysis. Comparison of demographic characteristics used for matching in TLR5-deficient and -sufficient women is depicted in Table S2. We used two-sided *t* tests to assess differences in bacterial colonization between TLR5-deficient and -sufficient study participants.

### Host response to flagellated BVAB.

Nonparametric Mann-Whitney tests were used for pairwise tests in HEK reporter cell experiments. Within cell types (HEK Null1 versus HEK hTLR5), comparisons were made between each experimental condition and the negative control. Where there were significant differences between the experimental condition and negative control, a between-cell-type comparison was made. Cytokine concentrations were compared between TLR5-deficient and -sufficient participants using Mann-Whitney nonparametric tests.

### Data availability.

Cohort metadata, quantitative PCR, and cytokine data are available at https://osf.io/tq9cf/?view_only=310002af29c54739abbee56dec8def9a. R scripts used for data analysis and figure generation are available at https://github.com/ejdelacruz/tlr5-deficiency-flagellated-vaginal-bacteria. Scripts used for processing metagenomic reads are available at https://github.com/SycuroLab/delaCruz_flaA_TLR5. Metagenomic reads have been uploaded to the NCBI Sequence Read Archive (SRA; study, PRJNA612150; sample, FHVM-1_L1H1 [SAMN14363795]; experiment, L1H1 [SRX7899168]; run, FHVM-1_L1H1_clean_R1.fastq [SRR11293606]).

## Supplementary Material

Supplemental file 1

## References

[1] Srinivasan S, Hoffman NG, Morgan MT, Matsen FA, Fiedler TL, Hall RW, Ross FJ, McCoy CO, Bumgarner R, Marrazzo JM, Fredricks DN. 2012. Bacterial communities in women with bacterial vaginosis: high resolution phylogenetic analyses reveal relationships of microbiota to clinical criteria. PLoS One 7:e37818. doi:10.1371/journal.pone.0037818. 22719852 PMC3377712

[2] Nugent RP, Krohn MA, Hillier SL. 1991. Reliability of diagnosing bacterial vaginosis is improved by a standardized method of gram stain interpretation. J Clin Microbiol 29:297–301. doi:10.1128/JCM.29.2.297-301.1991. 1706728 PMC269757

[3] De Boer JM, Plantema FH. 1988. Ultrastructure of the in situ adherence of Mobiluncus to vaginal epithelial cells. Can J Microbiol 34:757–766. doi:10.1139/m88-129. 3203257

[4] Spiegel CA, Eschenbach DA, Amsel R, Holmes KK. 1983. Curved anaerobic bacteria in bacterial (nonspecific) vaginosis and their response to antimicrobial therapy. J Infect Dis 148:817–822. doi:10.1093/infdis/148.5.817. 6631073

[5] Spiegel C. 1984. Mobiluncus curtisii and Mobiluncus mulieris, curved motile bacteria from the human vagina. Clin Microbiol Newsl 6:163–165. doi:10.1016/S0196-4399(84)80110-5.

[6] Srinivasan S, Morgan MT, Liu C, Matsen FA, Hoffman NG, Fiedler TL, Agnew KJ, Marrazzo JM, Fredricks DN. 2013. More than meets the eye: associations of vaginal bacteria with gram stain morphotypes using molecular phylogenetic analysis. PLoS One 8:e78633. doi:10.1371/journal.pone.0078633. 24302980 PMC3840219

[7] Fredricks DN, Fiedler TL, Marrazzo JM. 2005. Molecular identification of bacteria associated with bacterial vaginosis. N Engl J Med 353:1899–1911. doi:10.1056/NEJMoa043802. 16267321

[8] Fredricks DN, Fiedler TL, Thomas KK, Oakley BB, Marrazzo JM. 2007. Targeted PCR for detection of vaginal bacteria associated with bacterial vaginosis. J Clin Microbiol 45:3270–3276. doi:10.1128/JCM.01272-07. 17687006 PMC2045326

[9] Fettweis JM, Serrano MG, Brooks JP, Edwards DJ, Girerd PH, Parikh HI, Huang B, Arodz TJ, Edupuganti L, Glascock AL, Xu J, Jimenez NR, Vivadelli SC, Fong SS, Sheth NU, Jean S, Lee V, Bokhari YA, Lara AM, Mistry SD, Duckworth RA, Bradley SP, Koparde VN, Orenda XV, Milton SH, Rozycki SK, Matveyev AV, Wright ML, Huzurbazar SV, Jackson EM, Smirnova E, Korlach J, Tsai YC, Dickinson MR, Brooks JL, Drake JI, Chaffin DO, Sexton AL, Gravett MG, Rubens CE, Wijesooriya NR, Hendricks-Muñoz KD, Jefferson KK, Strauss JF, Buck GA. 2019. The vaginal microbiome and preterm birth. Nat Med 25:1012–1021. doi:10.1038/s41591-019-0450-2. 31142849 PMC6750801

[10] Nelson DB, Hanlon A, Nachamkin I, Haggerty C, Mastrogiannis DS, Liu C, Fredricks DN. 2014. Early pregnancy changes in bacterial vaginosis-associated bacteria and preterm delivery. Paediatr Perinat Epidemiol 28:88–96. doi:10.1111/ppe.12106. 24405280 PMC4031320

[11] Blackwell AL, Fox AR, Phillips I, Barlow D. 1983. Anaerobic vaginosis (non-specific vaginitis): clinical, microbiological, and therapeutic findings. Lancet ii:1379–1382. doi:10.1016/s0140-6736(83)90920-0. 6140492

[12] Smith KD, Andersen-Nissen E, Hayashi F, Strobe K, Bergman MA, Barrett SL, Cookson BT, Aderem A. 2003. Toll-like receptor 5 recognizes a conserved site on flagellin required for protofilament formation and bacterial motility. Nat Immunol 4:1247–1253. doi:10.1038/ni1011. 14625549

[13] Andersen-Nissen E, Smith KD, Strobe KL, Barrett SL, Cookson BT, Logan SM, Aderem A. 2005. Evasion of Toll-like receptor 5 by flagellated bacteria. Proc Natl Acad Sci U S A 102:9247–9252. doi:10.1073/pnas.0502040102. 15956202 PMC1166605

[14] Andersen-Nissen E, Smith KD, Bonneau R, Strong RK, Aderem A. 2007. A conserved surface on Toll-like receptor 5 recognizes bacterial flagellin. J Exp Med 204:393–403. doi:10.1084/jem.20061400. 17283206 PMC2118731

[15] Forstnerič V, Ivičak-Kocjan K, Plaper T, Jerala R, Benčina M. 2017. The role of the C-terminal D0 domain of flagellin in activation of Toll like receptor 5. PLoS Pathog 13:e1006574. doi:10.1371/journal.ppat.1006574. 28827825 PMC5578693

[16] Pioli PA, Amiel E, Schaefer TM, Connolly JE, Wira CR, Guyre PM. 2004. Differential expression of Toll-like receptors 2 and 4 in tissues of the human female reproductive tract. Infect Immun 72:5799–5806. doi:10.1128/IAI.72.10.5799-5806.2004. 15385480 PMC517561

[17] Fazeli A, Bruce C, Anumba DO. 2005. Characterization of Toll-like receptors in the female reproductive tract in humans. Hum Reprod 20:1372–1378. doi:10.1093/humrep/deh775. 15695310

[18] Hawn TR, Verbon A, Lettinga KD, Zhao LP, Li SS, Laws RJ, Skerrett SJ, Beutler B, Schroeder L, Nachman A, Ozinsky A, Smith KD, Aderem A. 2003. A common dominant TLR5 stop codon polymorphism abolishes flagellin signaling and is associated with susceptibility to legionnaires' disease. J Exp Med 198:1563–1572. doi:10.1084/jem.20031220. 14623910 PMC2194120

[19] Andersen-Nissen E, Hawn TR, Smith KD, Nachman A, Lampano AE, Uematsu S, Akira S, Aderem A. 2007. Cutting edge: Tlr5-/- mice are more susceptible to Escherichia coli urinary tract infection. J Immunol 178:4717–4720. doi:10.4049/jimmunol.178.8.4717. 17404249

[20] Hawn TR, Scholes D, Li SS, Wang H, Yang Y, Roberts PL, Stapleton AE, Janer M, Aderem A, Stamm WE, Zhao LP, Hooton TM. 2009. Toll-like receptor polymorphisms and susceptibility to urinary tract infections in adult women. PLoS One 4:e5990. doi:10.1371/journal.pone.0005990. 19543401 PMC2696082

[21] Meena NK, Ahuja V, Meena K, Paul J. 2015. Association of TLR5 gene polymorphisms in ulcerative colitis patients of north India and their role in cytokine homeostasis. PLoS One 10:e0120697. doi:10.1371/journal.pone.0120697. 25789623 PMC4366177

[22] Gewirtz AT, Vijay-Kumar M, Brant SR, Duerr RH, Nicolae DL, Cho JH. 2006. Dominant-negative TLR5 polymorphism reduces adaptive immune response to flagellin and negatively associates with Crohn's disease. Am J Physiol Gastrointest Liver Physiol 290:G1157–G1163. doi:10.1152/ajpgi.00544.2005. 16439468

[23] Koumans EH, Sternberg M, Bruce C, McQuillan G, Kendrick J, Sutton M, Markowitz LE. 2007. The prevalence of bacterial vaginosis in the United States, 2001-2004; associations with symptoms, sexual behaviors, and reproductive health. Sex Transm Dis 34:864–869. doi:10.1097/OLQ.0b013e318074e565. 17621244

[24] Jacchieri SG, Torquato R, Brentani RR. 2003. Structural study of binding of flagellin by Toll-like receptor 5. J Bacteriol 185:4243–4247. doi:10.1128/jb.185.14.4243-4247.2003. 12837800 PMC164893

[25] Kelley LA, Mezulis S, Yates CM, Wass MN, Sternberg MJ. 2015. The Phyre2 web portal for protein modeling, prediction and analysis. Nat Protoc 10:845–858. doi:10.1038/nprot.2015.053. 25950237 PMC5298202

[26] Muzny CA, Sunesara IR, Griswold ME, Kumar R, Lefkowitz EJ, Mena LA, Schwebke JR, Martin DH, Swiatlo E. 2014. Association between BVAB1 and high Nugent scores among women with bacterial vaginosis. Diagn Microbiol Infect Dis 80:321–323. doi:10.1016/j.diagmicrobio.2014.09.008. 25262105 PMC4326426

[27] Nasu K, Narahara H. 2010. Pattern recognition via the toll-like receptor system in the human female genital tract. Mediators Inflamm 2010:976024. doi:10.1155/2010/976024. 20396665 PMC2853082

[28] Ali ASM, Mowbray C, Lanz M, Stanton A, Bowen S, Varley CL, Hilton P, Brown K, Robson W, Southgate J, Aldridge PD, Tyson-Capper A, Abraham S, Pickard RS, Hall J. 2017. Targeting deficiencies in the TLR5 mediated vaginal response to treat female recurrent urinary tract infection. Sci Rep 7:11039. doi:10.1038/s41598-017-10445-4. 28887442 PMC5591273

[29] Duncan JA, Canna SW. 2018. The NLRC4 inflammasome. Immunol Rev 281:115–123. doi:10.1111/imr.12607. 29247997 PMC5897049

[30] Uversky VN. 2015. Functional roles of transiently and intrinsically disordered regions within proteins. FEBS J 282:1182–1189. doi:10.1111/febs.13202. 25631540

[31] Uversky VN. 2013. A decade and a half of protein intrinsic disorder: biology still waits for physics. Protein Sci 22:693–724. doi:10.1002/pro.2261. 23553817 PMC3690711

[32] Barker CS, Meshcheryakova IV, Kostyukova AS, Freddolino PL, Samatey FA. 2017. An intrinsically disordered linker controlling the formation and the stability of the bacterial flagellar hook. BMC Biol 15:97. doi:10.1186/s12915-017-0438-7. 29078764 PMC5660449

[33] Oguri T, Kwon Y, Woo JKK, Prehna G, Lee H, Ning M, Won K-J, Lee J, Mei S, Shi Y, Jeong H, Lee H. 2018. A family of small intrinsically disordered proteins involved in flagellum-dependent motility in Salmonella enterica. J Bacteriol 201:e00415-18. doi:10.1128/JB.00415-18. 30373755 PMC6304668

[34] Muzny CA, Blanchard E, Taylor CM, Aaron KJ, Talluri R, Griswold ME, Redden DT, Luo M, Welsh DA, Van Der Pol WJ, Lefkowitz EJ, Martin DH, Schwebke JR. 2018. Identification of key bacteria involved in the induction of incident bacterial vaginosis: a prospective study. J Infect Dis 218:966–978. doi:10.1093/infdis/jiy243. 29718358 PMC6093354

[35] Srinivasan S, Liu C, Mitchell CM, Fiedler TL, Thomas KK, Agnew KJ, Marrazzo JM, Fredricks DN. 2010. Temporal variability of human vaginal bacteria and relationship with bacterial vaginosis. PLoS One 5:e10197. doi:10.1371/journal.pone.0010197. 20419168 PMC2855365

[36] Srinivasan S, Morgan MT, Fiedler TL, Djukovic D, Hoffman NG, Raftery D, Marrazzo JM, Fredricks DN. 2015. Metabolic signatures of bacterial vaginosis. mBio 6:e00204-15. doi:10.1128/mBio.00204-15. 25873373 PMC4453549

[37] Wattam AR, Davis JJ, Assaf R, Boisvert S, Brettin T, Bun C, Conrad N, Dietrich EM, Disz T, Gabbard JL, Gerdes S, Henry CS, Kenyon RW, Machi D, Mao C, Nordberg EK, Olsen GJ, Murphy-Olson DE, Olson R, Overbeek R, Parrello B, Pusch GD, Shukla M, Vonstein V, Warren A, Xia F, Yoo H, Stevens RL. 2017. Improvements to PATRIC, the all-bacterial Bioinformatics Database and Analysis Resource Center. Nucleic Acids Res 45:D535–D542. doi:10.1093/nar/gkw1017. 27899627 PMC5210524

[38] Kearse M, Moir R, Wilson A, Stones-Havas S, Cheung M, Sturrock S, Buxton S, Cooper A, Markowitz S, Duran C, Thierer T, Ashton B, Meintjes P, Drummond A. 2012. Geneious Basic: an integrated and extendable desktop software platform for the organization and analysis of sequence data. Bioinformatics 28:1647–1649. doi:10.1093/bioinformatics/bts199. 22543367 PMC3371832

[39] Rossez Y, Wolfson EB, Holmes A, Gally DL, Holden NJ. 2015. Bacterial flagella: twist and stick, or dodge across the kingdoms. PLoS Pathog 11:e1004483. doi:10.1371/journal.ppat.1004483. 25590430 PMC4295861

[40] DSMZ. 2020. 104. PYG medium (modified). List of media for microorganisms. Deutsche Sammlung von Mikroorganismen und Zellkulturen GmbH. https://www.dsmz.de/collection/catalogue/microorganisms/culture-technology/list-of-media-for-microorganisms.

