# Supplementary material for: Genetic Variation in Toll-Like Receptor 5 and Colonization with Flagellated Bacterial Vaginosis-Associated Bacteria
Source: Infect Immun. 2021 Feb 16;89(3):e00060-20. doi: 10.1128/IAI.00060-20 (PMC8097276; doi:10.1128/IAI.00060-20)
Supplement: Supplemental file 1 [file IAI.00060-20-s0001.pdf]

Supplementary Material

Supplementary Tables

Table S1. Discovery cohort baseline characteristics by TLR5 rs5744168 genotype resolution

	TLR5 rs5744168 Genotype Resolved N = 189		TLR5 rs5744168 Genotype Unresolved N = 24		Total N = 213		
	N	%	N	%	N	%	p_{χ^2}
Nugent Score							
0 - 3	88	47%	6	25%	94	44%	0.07
4 - 6	23	12%	6	25%	29	14%	
7 - 10	78	41%	12	50%	90	42%	
Race							
AIAN/NHPI[a]	7	4%	0	0%	7	3%	0.03
Asian	3	2%	1	4%	4	2%	
African-American	60	32%	14	58%	74	35%	
Caucasian	103	54%	8	33%	111	52%	
Other	15	8%	0	0%	15	7%	
Two or more races	1	1%	1	4%	2	1%	
Ethnicity							
Hispanic	13	7%	2	8%	15	7%	0.53
Non-Hispanic	152	80%	20	83%	172	81%	
Refuse to report	24	13%	2	8%	26	12%	
Age							
18-30	90	48%	12	50%	102	48%	0.27
31-40	54	29%	3	13%	57	27%	
41-50	42	22%	8	33%	50	23%	
Refuse to report	3	2%	1	4%	4	2%	
Hormonal Contraception							
Yes	63	33%	9	38%	72	34%	0.86
No	126	67%	15	63%	141	66%	
Refuse to report	0	0%	0	0%	0	0%	
History of BV							
Yes	133	70%	15	63%	148	69%	0.71
No	51	27%	8	33%	59	28%	
Refuse to report	5	3%	1	4%	6	3%	

[a]American Indian, Alaska Native, Native Hawaiian, Pacific Islander

Table S2. Validation cohort - cases/controls analyzed for bacterial colonization

	TLR5 rs5744168	
	CT/TT	CC
	Deficient (Cases)	Sufficient (Controls)
N	8	32
Age (years, average)	33.9	33.1
% Caucasian	75.0%	75.0%
% Hormonal contraception	0.0%	0.0%
% Vaginal douching (ever)	75.0%	50.0%

Table S3. Cohort 3, colonization with flagellated BV-associated bacteria

	16S rRNA Copies Per Swab (by qPCR)		
Sample Name	*Mobiluncus curtisii*	*Mobiluncus mulieris*	BVAB1
C3A	9.4E+01	8.9E+07	1.41E+09
C3B	9.4E+01	1.4E+05	3.55E+09
C3C	6.2E+04	1.9E+07	1.34E+10
C3D	9.4E+01	6.4E+05	2.00E+09
C3E	2.3E+07	2.2E+04	9.34E+09
C3F	5.6E+03	1.1E+08	2.37E+09
C3G	2.9E+05	3.9E+02	3.00E+09
C3H	9.4E+02	2.5E+07	6.60E+09
C3I	6.9E+05	1.1E+08	4.44E+09
C3J	4.1E+06	2.1E+06	1.00E+10
C3K	9.4E+01	1.3E+07	9.76E+08
C3L	1.5E+04	5.8E+06	1.73E+09
C3M	9.4E+01	8.6E+05	9.76E+08
C3N	8.7E+04	1.4E+08	5.47E+09
C3O	9.4E+01	2.5E+02	2.68E+05
C3P	3.8E+05	7.6E+06	4.50E+09
C3Q	3.4E+06	1.0E+08	4.59E+09

Supplementary Figures

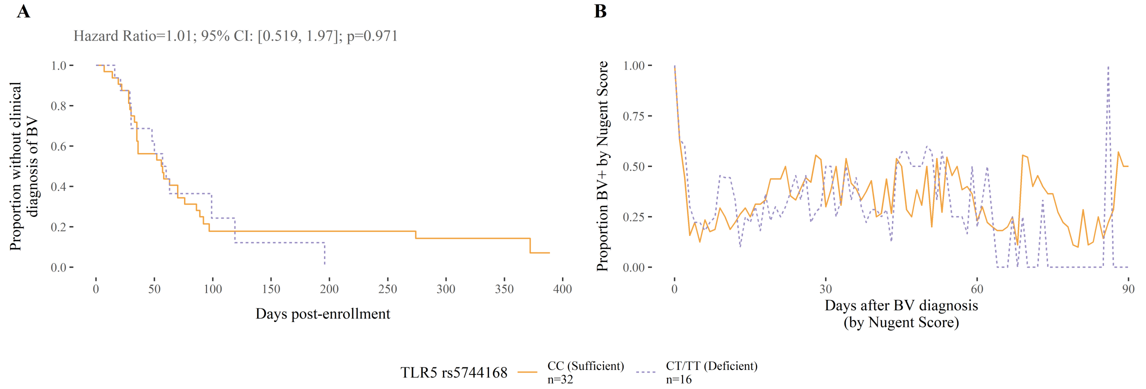

C

	TLR5 rs5744168 CC (Sufficient)	TLR5 rs5744168 CT/TT (Deficient)
N	32	16
% Nugent+ at Enrollment	65.6	56.2
Person-Days Observed	2240	933
% Days Nugent+	20.7	22.6
Age (Mean)	30.2	30.4
% African American	50	25
% WSM	53.1	62.5
% WSW	15.6	25
% Hormonal Contraception	40.6	37.5

Figure S1. TLR5 deficient cases and TLR5 sufficient controls in the discovery cohort have similar risk of BV.

TLR5 deficient cases and TLR5 sufficient controls were matched on Nugent diagnosis at enrollment, age, race, sexual preference, and hormonal contraception use in a 2:1 ratio. (**A**) Survival curves depicting time to first diagnosis with BV by Amsel's criteria, stratified by TLR5 deficiency. There are no significant differences in time to first BV diagnosis between TLR5 sufficient controls and TLR5 deficient cases. (**B**) Daily average proportion of women who are BV-positive by Nugent score, stratified by TLR5 genotype, starting at the time of first Nugent diagnosis of BV, and following up to 100 days post-diagnosis. (**C**) Demographic characteristics of TLR5 sufficient and deficient women from longitudinal cohort included in analysis of bacterial colonization. Groups appear balanced for major risks of BV diagnosis, including BV diagnosis at enrollment, age, race, and sexual behaviors (women who have sex with men [WSM], women who have sex with women [WSW]), and hormonal contraceptive status.

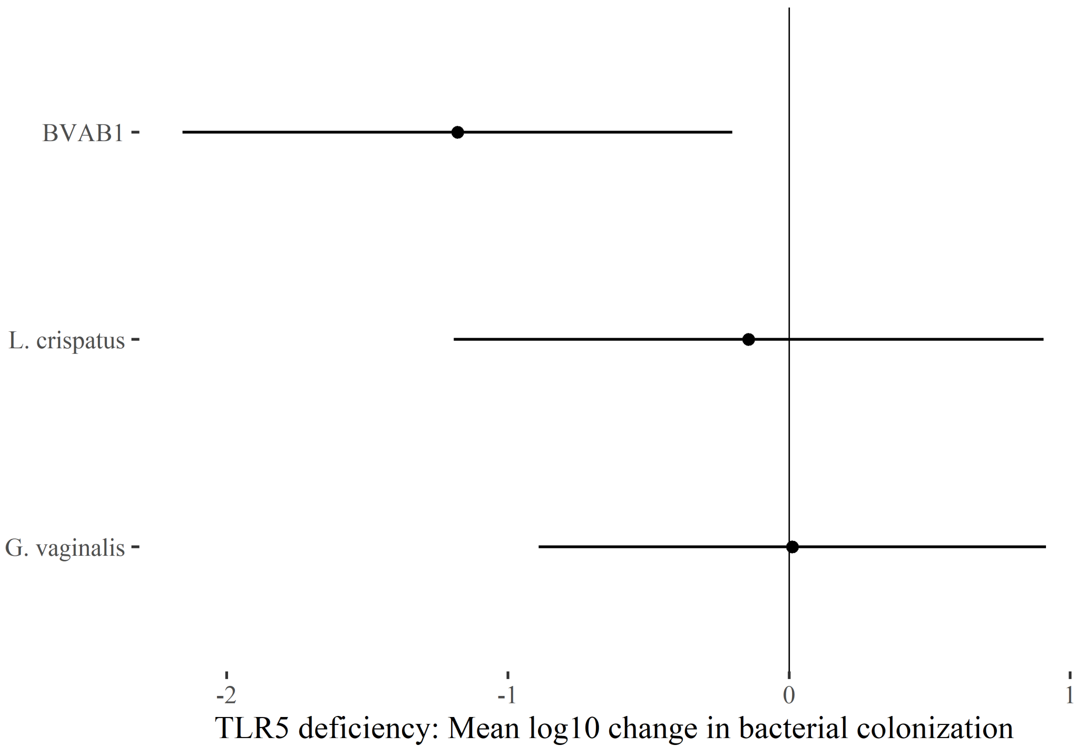

Figure S2. TLR5 deficiency is associated with decreased colonization with BVAB1, but is not associated with differences in colonization with *Gardnerella vaginalis* or *Lactobacillus crispatus*.

Dots depict $\log_{10}$ point estimate of difference in bacterial concentration in TLR5 deficient women (compared to TLR5 sufficient women). Horizontal lines represent 95% confidence intervals of each estimate. Vertical line denotes zero, or no difference in bacterial colonization between TLR5 deficient and sufficient women; 95% confidence intervals intersecting this vertical line represent species where data does not suggest a statistically significant difference.

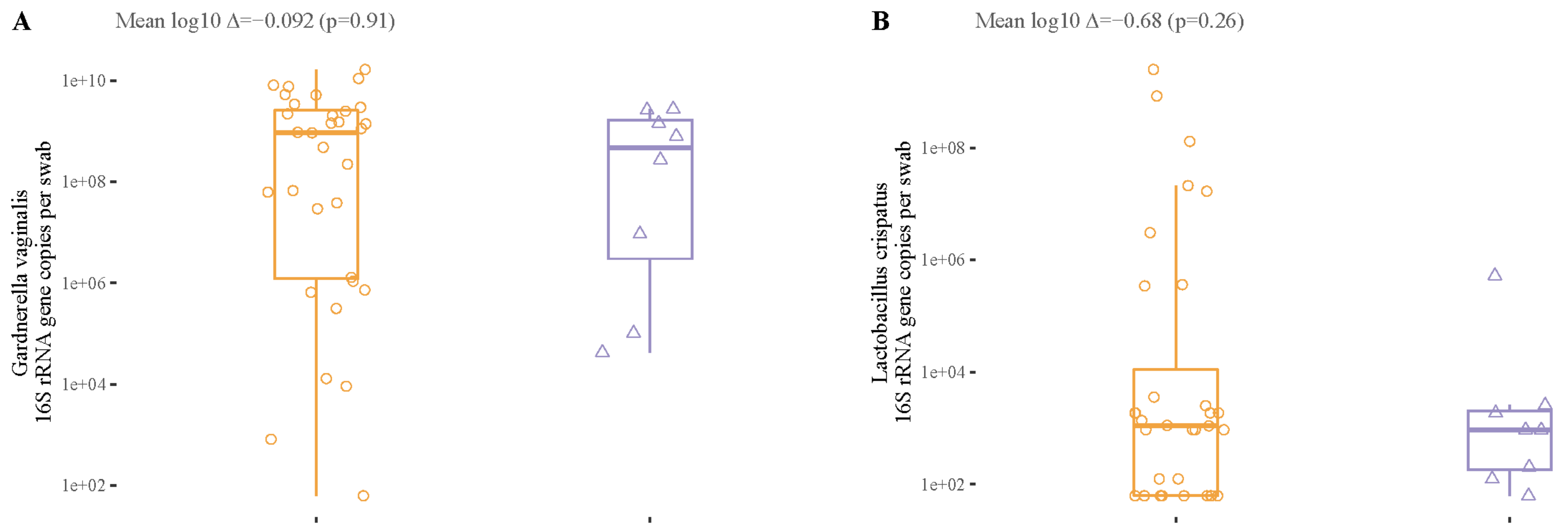

Figure S3. TLR5 deficiency does not modify colonization of *Gardnerella vaginalis* or *Lactobacillus crispatus*. Estimated bacterial concentration (by species-specific 16S rRNA gene qPCR) in individual study participants represented in scatterplots, with overlying boxplots to summarize overall distribution. Comparisons between TLR5 deficient and sufficient groups performed using two-sided, Student's t-test.

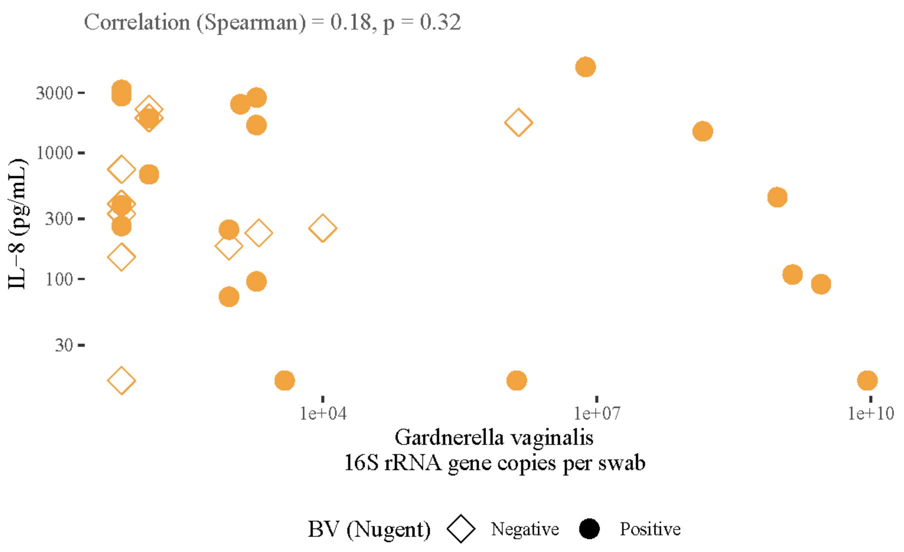

Figure S4. IL-8 concentration does not correlate with *Gardnerella vaginalis* colonization in TLR5 sufficient women.

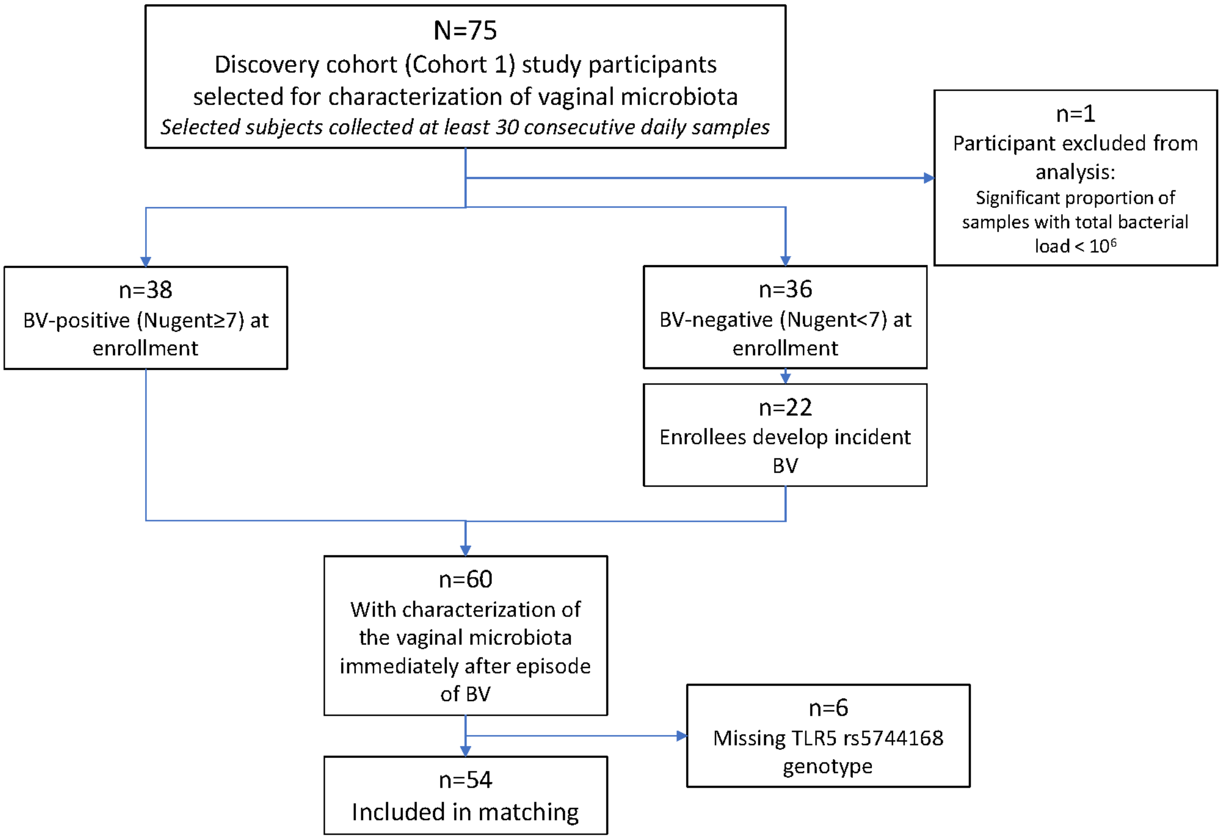

Figure S5. Discovery cohort (Cohort 1) study enrollees observations included for analysis.
